# Halophilic plant growth-promoting bacterial consortium reshapes soil microbiota to enhance salinity tolerance, antioxidant defense, and yield in *Vigna mungo* L.

**DOI:** 10.3389/fmicb.2026.1878014

**Published:** 2026-08-11

**Authors:** Daniel Raphael, Theivasigamani Parthasarathi

**Affiliations:** VIT School of Agricultural Innovations and Advanced Learning (VAIAL), Vellore Institute of Technology (VIT), Vellore, Tamil Nadu, India

**Keywords:** antioxidant enzymes, HPGPB, microbial consortium, salinity stress, *Vigna mungo* L.

## Abstract

Soil salinity is a major abiotic stress that severely restricts crop productivity by disrupting ionic balance, inducing osmotic stress, and promoting oxidative damage. Black gram (*Vigna mungo* L.), an important pulse crop, is highly sensitive to salinity, resulting in reduced growth, physiological performance, and yield. The present study evaluated the efficacy of a compatible multi-strain HPGPB consortium comprising MKM3 (*Halobacillus marinus*), MKM4 (*Halobacillus halophilus*), and MKM11 (*Halobacillus halophilus*) in enhancing salinity tolerance in two black gram varieties (VBN8 and VBN11) under greenhouse conditions. Plants were subjected to 50 and 100 mM NaCl stress, with and without consortium inoculation, in a completely randomized design. Salinity stress significantly reduced plant growth, photosynthetic pigments, biomass, nutrient uptake, and grain yield, while increasing Na^+^ accumulation, lipid peroxidation, and osmotic stress markers. Consortium inoculation effectively mitigated these adverse effects by improving plant height, root development, biomass, and grain yield by up to 46 and 38%, respectively, under saline conditions. Consortium-inoculated plants exhibited improved photosynthetic performance, enhanced nutrient uptake and ionic balance, reduced Na^+^ accumulation and malondialdehyde content, and increased activities of antioxidant enzymes, indicating enhanced salinity tolerance. Among the tested varieties, VBN11 exhibited greater salinity tolerance and a stronger response to consortium inoculation than VBN8. Rhizosphere metagenomic analysis revealed consortium-associated shifts in microbial community structure under saline conditions. Collectively, the results demonstrate that the HPGPB consortium enhances salinity tolerance through coordinated physiological, biochemical, and microbiome-associated mechanisms. These findings highlight the potential of HPGPB consortia as sustainable bioinoculants for improving black gram productivity in salt-affected agroecosystems.

## Graphical Abstract

**Figure:**
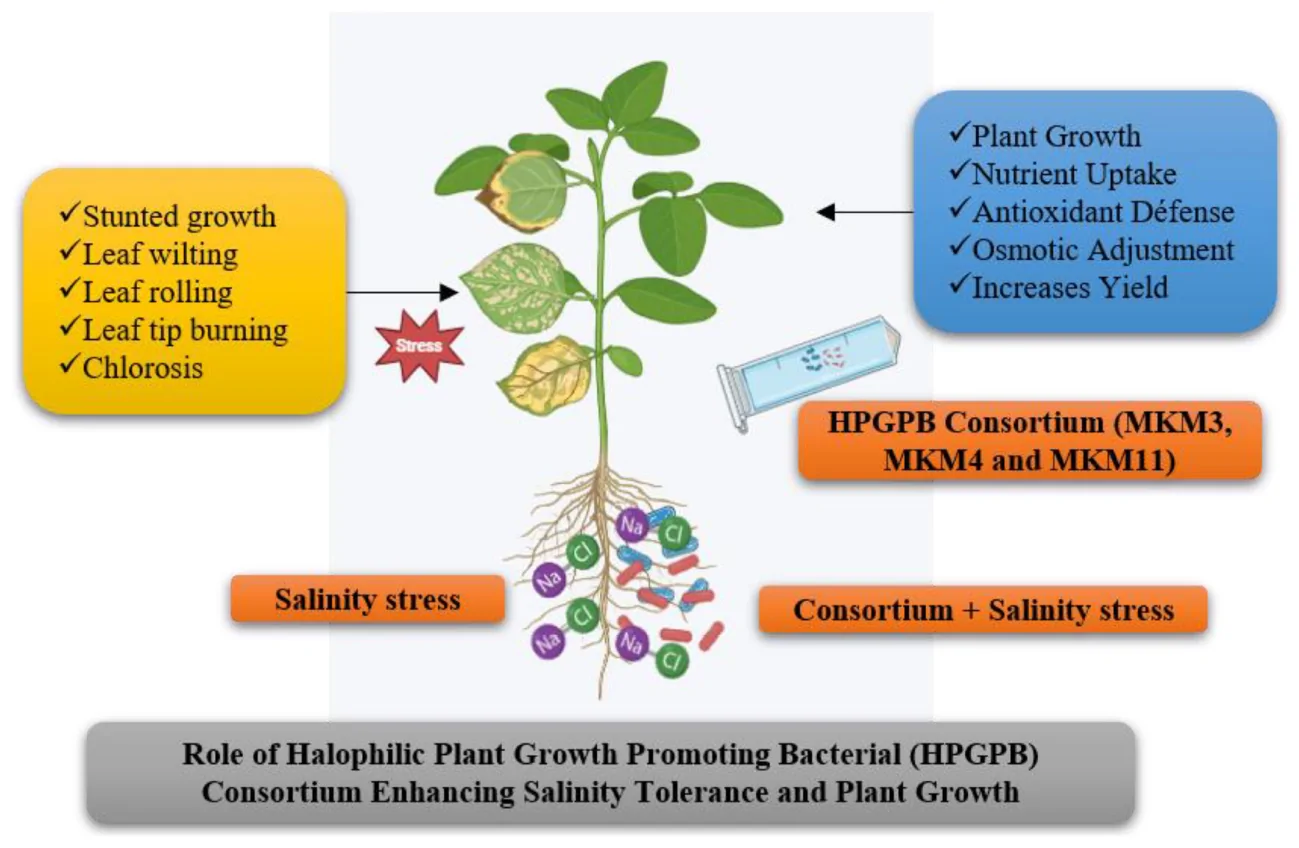
Schematic representation of the mechanisms by which the halophilic plant growth-promoting bacterial (HPGPB) consortium (MKM3, MKM4, and MKM11) mitigates salinity stress by improving nutrient uptake, antioxidant defence, osmotic adjustment, plant growth, and yield while reducing salt-induced damage.

## Highlights

1. HPGPB consortium enhanced salinity tolerance in black gram.
2. Consortium inoculation reduced Na^+^ accumulation under salt stress conditions.
3. Consortium-treated plants showed improved nutrient uptake and ionic homeostasis.
4. HPGPB enhanced antioxidant enzyme activities and mitigated oxidative stress.
5. Consortium inoculation significantly improved biomass and grain yield under salinity stress.

## 1 Introduction

Salinity is one of the most critical abiotic stresses limiting agricultural productivity worldwide. It adversely affects plant physiology and development, leading to considerable yield losses. Approximately 1.5 billion hectares of land worldwide are affected by soil degradation, a substantial proportion of which is influenced by salinization (Singh and Prasad, 2020; Kumawat et al., 2023). Globally, around 20% of irrigated agricultural land is already affected by salt accumulation (Singh, 2022; Patel et al., 2023). The rising soil salinity continues to diminish crop productivity and threatens global food security (Khan et al., 2021; Mukhopadhyay et al., 2021; Gulzar et al., 2025). Elevated salinity interferes with plant germination, growth, and metabolic functions, ultimately reducing both yield and quality (Van Zelm et al., 2020; Fotoohiyan et al., 2024; Passa et al., 2026).

Excessive soluble salts in the rhizosphere induce salinity stress, which disrupts plant metabolism through two primary mechanisms (Khan et al., 2021; Kalam et al., 2020). High salt concentrations create osmotic and ionic imbalances, damaging root tissues and limiting water and nutrient uptake. This disruption further induces oxidative stress, generating reactive oxygen species (ROS) that damage cell membranes, proteins, and nucleic acids. The resulting lipid peroxidation and DNA degradation destabilize cellular integrity and can activate programmed cell death pathways, causing irreversible physiological damage (Zhang et al., 2024; Seleiman et al., 2022).

Halophilic microorganisms, often termed “halophiles,” constitute a unique group of extremophiles capable of thriving in environments with elevated salt concentrations such as salt pans, saline soils, hypersaline lakes, and solar salterns. These microbes possess specialized physiological and molecular mechanisms, including the accumulation of compatible solutes (e.g., ectoine, glycine betaine) and the regulation of ion homeostasis, which allow them to sustain metabolic activity under osmotic stress (Meinzer et al., 2023; Orhan, 2021; Rathod et al., 2023; Lim et al., 2005). Recently, HPGPB have emerged as a promising biological tool to alleviate salinity-induced stress in plants (Tanur Erkoyuncu et al., 2026; Yan et al., 2024). These salt-tolerant bacteria facilitate plant growth through several mechanisms, including the synthesis of phytohormones such as indole-3-acetic acid (IAA), which promotes root elongation and cell division, and the production of osmoprotectants (trehalose, glycerol) that help to maintain cell turgor pressure and protect cellular structures. Additionally, they secrete siderophores that enhance iron acquisition, solubilize phosphate and potassium in soil, produce exopolysaccharides that improve soil aggregation and water retention, and release enzymes that activate plant antioxidant defense systems, reducing ROS toxicity. HPGPB also regulate ionic balance by modulating Na^+^ and K^+^ uptake, mitigating ion toxicity, and may induce systemic resistance against abiotic and biotic stresses (Meinzer et al., 2023; AbuQamar et al., 2024; Ould Ouali et al., 2024; Sridhar et al., 2025a; Jose and Sankar, 2025).

Numerous halophilic and halotolerant bacterial species have been reported to enhance plant performance in saline environments. For instance, *Bacillus haynesii* enhances soil nutrient availability and produces IAA, thereby stimulating plant growth under saline–alkaline conditions (Yue et al., 2022; Pérez-Inocencio et al., 2022). Similarly, *Pseudomonas fluorescens* produces siderophores and IAA that support nutrient uptake and stress adaptation in plants (Kumawat et al., 2024). *Enterobacter cloacae* and *Klebsiella pneumoniae* exhibit phosphate solubilization, nitrogen fixation, and IAA production, improving plant growth in salt-stressed soils (Ji et al., 2020; Khumairah et al., 2022; Pérez-Inocencio et al., 2022). Other halophilic genera, including *Oceanobacillus aidingensis*, *Arhodomonas aquaeolei*, *Brachybacterium saurashtrense*, and *Chryseobacterium oleae*, have demonstrated significant plant growth-promoting activities such as exopolysaccharide production, IAA synthesis, and effective root colonization, highlighting their potential role in sustainable agriculture within saline ecosystems (Gontia et al., 2011; Meza et al., 2022; Oliva et al., 2023; Kumar et al., 2020, 2023).

Black gram (*Vigna mungo* L.) is an important pulse crop extensively cultivated in tropical and subtropical regions, particularly across South and Southeast Asia. It serves as a valuable source of dietary protein (22–26%), carbohydrates, and essential minerals, contributing significantly to global nutritional security. Beyond its nutritional importance, black gram improves soil fertility through symbiotic biological nitrogen fixation, thereby playing a vital role in sustainable cropping systems (Nair et al., 2024).

Despite its agronomic significance, black gram productivity is markedly constrained by salinity stress, especially in salt-affected soils and regions dependent on saline irrigation. The crop is moderately sensitive to salinity, and elevated salt levels negatively influence seed germination, vegetative development, and yield. Salinity disrupts critical physiological and biochemical processes, including photosynthesis, nutrient uptake, and nodulation efficiency. Elevated concentrations of Na^+^ and Cl^−^ ions in the root zone adversely affect osmotic regulation, resulting in ionic imbalance, nutrient deficiency, and reduced plant growth. Consequently, affected plants exhibit chlorosis, reduced nodulation, impaired nitrogen fixation, and stunted growth (Adlinge et al., 2014; Shanthi et al., 2021). Furthermore, salinity-induced disruption of Na^+^/K^+^ homeostasis interferes with enzymatic functions and photosynthetic efficiency, thereby further limiting plant growth and productivity (Thadani et al., 2023).

Considering these constraints, black gram serves as an ideal model crop for evaluating strategies to mitigate salinity stress. In this context, HPGPB have emerged as a sustainable and effective approach for enhancing plant tolerance under saline conditions. However, information on the synergistic effects of multi-strain HPGPB consortia in black gram under varying salinity levels remains limited. Moreover, comprehensive studies integrating physiological, biochemical, nutritional, and rhizosphere microbial responses to consortium inoculation are scarce, and genotype-specific responses have not been thoroughly investigated. Therefore, the present study evaluates a compatible multi-strain HPGPB consortium for enhancing salinity tolerance in two black gram varieties through the coordinated regulation of antioxidant defense, photosynthetic efficiency, nutrient acquisition, and rhizosphere microbial dynamics.

## 2 Materials and methods

### 2.1 Isolation and characterization

Soil samples were collected from the Marakkanam salt pan, Tamil Nadu, India (12.19983° N, 79.9574° E), and halophilic bacteria were isolated on Halophilic Agar (HA; HiMedia Laboratories Pvt. Ltd., Mumbai, India) by the serial dilution and spread plate technique. The HA medium consisted of (g L^−1^): Acicase™ (10.0), yeast extract (10.0), proteose peptone (5.0), sodium citrate (3.0), potassium chloride (2.0), magnesium sulfate (25.0), sodium chloride (250.0), and agar (20.0), with a final pH of 7.2 ± 0.2 (at 25 °C). Inoculated plates were incubated at 30 ± 2 °C for up to 12 days, and morphologically distinct colonies were purified through repeated streaking to obtain pure cultures. Salt tolerance was assessed on HA medium supplemented with 2.5–25% (w/v) NaCl to evaluate bacterial growth across a broad salinity gradient and identify highly salt-tolerant isolates for subsequent studies.

### 2.2 Molecular identification of isolates

Genomic DNA was extracted from selected bacterial isolates, and the 16S rRNA gene was amplified using universal bacterial primers by PCR amplification. The amplified products were sequenced bidirectionally using Sanger sequencing. Raw sequences were quality-checked, assembled into consensus sequences, and compared with reference sequences in the National Centre for Biotechnology Information (NCBI) database using the BLASTn tool. Multiple sequence alignment was performed using MEGA version 11. Phylogenetic analysis was conducted using the Neighbor-Joining method with the Kimura two-parameter model, and statistical confidence was evaluated using 1,000 bootstrap replicates. Species identity was assigned based on ≥ 99% sequence similarity and clustering with reference strains. The representative isolates were identified as *Halobacillus marinus* (MKM3; GenBank accession no. PP733532) and *Halobacillus halophilus* (MKM4; PP733534; MKM11; PP737116) (Janda and Abbott, 2007).

### 2.3 *In vitro* evaluation of plant growth-promoting traits

For ammonia production, bacterial isolates were inoculated into peptone water (20.0 g L^−1^ peptone; HiMedia, India) containing NaCl (30.0 g L^−1^; HiMedia, India) and incubated at 30 ± 2 °C for 48 h with shaking at 140 rpm. After incubation, 1 mL of Nessler’s reagent was added. Development of a yellow to dark brown coloration indicated positive ammonia production (Kapadia et al., 2022).

For indole-3-acetic acid (IAA) production, isolates were grown in nutrient broth (HiMedia, India) supplemented with L-tryptophan (0.1 mg mL^−1^) and incubated at 30 ± 2 °C for 48 h. Cultures were centrifuged at 8,000 rpm for 10 min, and 1 mL of supernatant was mixed with 2 mL of Salkowski’s reagent (0.5 M FeCl_3_ in 35% perchloric acid). After incubation in the dark for 30 min, pink to brown coloration indicated IAA production, and absorbance was recorded at 535 nm (Kapadia et al., 2022).

Phosphate solubilization was assessed on Pikovskaya’s agar medium (HiMedia, India) by incubating plates at 30 ± 2 °C for 7 days, and clear halo zones around colonies indicated positive activity (Albdaiwi et al., 2019). Potassium solubilization was evaluated using Aleksandrov agar medium (HiMedia, India) under similar incubation conditions, with halo zone formation indicating activity (Jha et al., 2011). Siderophore production was determined using Chrome Azurol S (CAS) agar medium, where orange-yellow halo formation around colonies indicated positive production (Kumar et al., 2024).

### 2.4 Selection of consortium and compatibility testing

Based on salt tolerance, multi-trait PGP activity, and screening performance, six representative isolates were shortlisted. Among these, MKM3 (*Halobacillus marinus*), MKM4 (*Halobacillus halophilus*), and MKM11 (*Halobacillus halophilus*) exhibited superior performance and were selected for consortium development. Compatibility among selected isolates was evaluated using a modified cross-streak assay (Raja et al., 2006) on Halophilic Agar plates in pairwise and three-strain combinations. Plates were incubated at 30 ± 2 °C and observed for inhibition zones, growth suppression, or morphological changes at interaction interfaces. Absence of antagonism and uninterrupted growth were considered indicators of compatibility. All assays were performed in triplicate. Based on compatibility results, previously reported PGP traits, and salinity tolerance (Raphael and Parthasarathi, 2026), the selected strains were formulated into a compatible consortium (Table 1).

**TABLE 1:** *In vitro* salt tolerance of the selected halophilic bacterial strains.

NaCl (% w/v)	MKM3 (*Halobacillus marinus*)	MKM4 (*Halobacillus halophilus*)	MKM11 (*Halobacillus halophilus*)
2.5	+	+	+
5	++	++	++
10	+++	+++	+++
15	++	+	++
20	–	–	+
25	–	–	–

Growth scoring: (–) No growth; (+) Slight growth; (++) Moderate growth; (+++) Strong growth.

### 2.5 Greenhouse experiment

A controlled greenhouse experiment was conducted at the Vellore Institute of Technology (VIT), Vellore, Tamil Nadu, India (12.968575° N, 79.161821° E), from June to August 2025 to evaluate the growth-promoting potential of the HPGPB consortium under saline stress conditions. The potted plants were arranged in a completely randomized design (CRD) with six treatments and five biological replicates per treatment. The treatments consisted of:

*T1*, control without bacterial inoculation and NaCl
*T2*, plants exposed to 50 mM NaCl without bacterial inoculation
*T3*, plants exposed to 100 mM NaCl without bacterial inoculation
*T4*, plants inoculated with the bacterial consortium under non-saline conditions
*T5*, consortium-inoculated plants subjected to moderate salinity (50 mM NaCl)
*T6*, consortium-inoculated plants subjected to high salinity stress (100 mM NaCl).

Seeds of *Vigna mungo* L. (black gram) cultivars VBN8 and VBN11 were obtained from an authorized seed supplier in Vellore, Tamil Nadu, India. Seeds were surface-sterilized with 0.1% (w/v) mercuric chloride for 1 min, rinsed five times with sterile double-distilled water, and air-dried under aseptic conditions (Vincent, 1970). Ten surface-sterilized seeds were sown in 10-inch rectangular plastic pots containing a sterilized soil mixture of sandy soil, red soil, and farmyard manure (1:1:1, v/v/v). The soil mixture was autoclaved at 121 °C and 15 psi for 60 min in a single autoclaving cycle before use. Each pot was considered one biological replicate (experimental unit), and each treatment consisted of five independent pots (*n* = 5). Following germination, 7–8 healthy plants were maintained per pot under uniform growth conditions. For growth, physiological, biochemical, and yield assessments, five randomly selected plants were sampled from each pot, and the mean value per pot was used for statistical analysis. Plants were maintained under natural daylight conditions (approximately 10–12 h day length), with an average temperature of 28 ± 2 °C and relative humidity of 60–70%.

Salinity stress was imposed by irrigating the pots with 50 mM NaCl (electrical conductivity, 5.4 ± 0.2 dS m^−1^) or 100 mM NaCl (10.7 ± 0.2 dS m^−1^) solutions. The target salinity levels were maintained throughout the experimental period by regular irrigation with the respective saline solutions. Based on their plant growth-promoting traits, salinity tolerance, and compatibility assay results, isolates MKM3, MKM4, and MKM11 were selected for consortium development. The consortium was prepared immediately before inoculation by mixing actively growing cultures of the three isolates in equal proportions (1:1:1, v/v/v).

The bacterial consortium was applied at 10, 25, and 45 days after sowing (DAS), corresponding to the seedling, vegetative, and flowering stages, using the soil-trenching method. Each pot received 2 mL of consortium suspension containing 5 × 10^8^ CFU mL^−1^. At the pod formation stage (55 DAS), the third fully expanded leaf from each plant was collected for physiological and biochemical analyses. At maturity, plants were harvested and evaluated for growth and yield parameters, including plant height, root length, leaf area, fresh and dry biomass, number of root nodules, grains per plant, and 100-grain weight. Plant samples collected at harvest were washed, oven-dried at 65 °C, and ground into a fine powder. Total nitrogen (N) was determined using the Kjeldahl method, while phosphorus (P), potassium (K), sodium (Na), calcium (Ca), magnesium (Mg), zinc (Zn), and iron (Fe) were analyzed after acid digestion. Phosphorus was measured by the vanadomolybdate colorimetric method, K and Na using a flame photometer, and Ca, Mg, Zn, and Fe using atomic absorption spectrophotometry (AAS), following standard procedures (Tandon, 2004; Behera, 2015).

### 2.6 Photosynthetic gas exchange measurement

Net photosynthetic rate, stomatal conductance, and transpiration rate were determined using a LI-6800 portable gas exchange system (LI-COR Biosciences, Lincoln, United States). Observations were conducted under saturating light conditions to evaluate peak photosynthetic performance. Uniform, fully expanded, and visually healthy leaves exposed to direct sunlight were chosen for all measurements to ensure consistency and minimize variability among experimental replicates (Tang et al., 2024).

### 2.7 Estimation of photosynthetic pigments

Fresh leaf tissue (0.5 g) was homogenized in 80% (v/v) acetone, and the extract was centrifuged at 10,000 rpm for 5 min at 4 °C. The clear supernatant was collected, and absorbance was recorded using a UV–visible spectrophotometer at 663 nm, 645 nm, and 480 nm by following standard protocols (Sridhar et al., 2025a,b). Pigment concentrations were expressed as mg g^−1^ fresh weight (FW) and calculated using the following equations:

Chlorophyll *a* (mg g^−1^) = (12.7 × A_663_) – (2.69 × A_645_)
Chlorophyll *b* (mg g^−1^) = (22.9 × A_645_) – (4.7 × A_663_)
Total chlorophyll (mg g^−1^) = (8.02 × A_663_) + (20.2 × A_645_)
Carotenoids (mg g^−1^) = [A_480_ + (0.114 × A_663_) – (0.638 × A_645_)]

### 2.8 Proline content

Proline concentration (μg g^−1^ fresh weight) was determined following the method described by Bates et al. (1973). Approximately 0.5 g fresh leaf tissue was homogenized in 3% sulfosalicylic acid. The homogenate was centrifuged at 4,000 rpm for 10 min, and the clear supernatant was collected for analysis. An equal volume of acid ninhydrin reagent was added to the sample, and the mixture was incubated in a water bath at 100 °C for 60 min to facilitate color development. The reaction was terminated by allowing the tubes to cool to room temperature. Subsequently, toluene was added, and the mixture was shaken for 15–30 s to extract the chromophore. The absorbance of the toluene phase was recorded at 520 nm using a spectrophotometer, with a reagent blank used for calibration.

### 2.9 Malondialdehyde content

Malondialdehyde (MDA) content was measured using the thiobarbituric acid (TBA) assay as described by Du and Bramlage (1992), with slight modifications. Fresh leaf tissue was homogenized thoroughly using a pre-chilled mortar and pestle. The homogenate was centrifuged at 4,000 rpm for 10 min, and the clear supernatant was collected for analysis. An aliquot (700 μL) of the ethanolic extract was mixed with an equal volume (700 μL) of a reaction solution containing 0.6% (w/v) TBA prepared in 10% (w/v) trichloroacetic acid (TCA). The mixture was incubated in a water bath at 95 °C for 25 min to promote the formation of MDA–TBA complexes. After incubation, the reaction was terminated by cooling the tubes to room temperature. The samples were then centrifuged at 11,000 rpm for 1 min to obtain a clear supernatant. Absorbance was measured at 532 nm and corrected for non-specific turbidity by subtracting the absorbance at 600 nm, using an appropriate reagent blank.

### 2.10 Antioxidant enzyme activity

Superoxide dismutase (SOD) activity was determined following the nitro blue tetrazolium (NBT) photoreduction assay described by Flohé and Otting (1984), with slight modifications. The total reaction volume of 2.5 mL comprised 50 μL of crude enzyme extract, 0.1 M potassium phosphate buffer (pH 7.8), 75 μM riboflavin, 2 mM NBT, 3 mM EDTA, and 13 mM methionine. The reaction mixture was exposed to light for 15 min to initiate photochemical reduction of NBT, after which the reaction was terminated. Absorbance was recorded at 560 nm using a spectrophotometer.

Peroxidase (POD) activity was determined according to the method of Kar and Mishra (1976), with slight modifications. Fresh plant tissue (100 mg) was homogenized in 10 mL of 0.1 M potassium phosphate buffer (pH 7.0) and centrifuged at 10,000 rpm for 10 min at 4 °C. The resulting supernatant was used as the enzyme extract. The reaction mixture (3.5 mL total volume) consisted of 0.5 mL of enzyme extract, 2.0 mL of 0.1 M potassium phosphate buffer (pH 7.0), 1.0 mL of 0.05 M pyrogallol, and 1.0 mL of 1% (v/v) hydrogen peroxide (H_2_O_2_). The addition of H_2_O_2_ initiated the reaction. The increase in absorbance due to the formation of purpurogallin was recorded at 420 nm for 1 min using a UV–visible spectrophotometer. A reaction mixture without enzyme extract served as the blank.

Catalase (CAT) activity was determined following the method of Chandlee and Scandalios (1984), with slight modifications. Fresh leaf tissue (100 mg) was homogenized in 10 mL of 0.1 M potassium phosphate buffer (pH 7.0) and centrifuged at 10,000 rpm for 15 min at 4 °C. The reaction mixture consisted of 0.4 mL enzyme extract, 2.6 mL phosphate buffer, and 0.4 mL H_2_O_2_. The decrease in absorbance due to H_2_O_2_ decomposition was monitored at 240 nm for 1 min at 25 °C. Catalase activity was expressed as U mg^−1^ protein.

Ascorbate peroxidase (APX) activity was assayed according to the method of Nakano and Asada (1981), with slight modifications. Fresh plant tissue (100 mg) was homogenized in 10 mL of 0.1 M potassium phosphate buffer (pH 7.0) containing 2 mM ascorbate and centrifuged at 10,000 rpm for 15 min at 4 °C. The reaction mixture (3 mL total volume) consisted of 50 mM potassium phosphate buffer (pH 7.0), 0.1 mM EDTA, 0.5 mM ascorbic acid, 0.1 mM H_2_O_2_, and 0.5 mL of enzyme extract. The decrease in absorbance due to ascorbate oxidation was monitored at 290 nm. Enzyme activity was expressed as U mg^−1^ protein.

Total soluble protein content was quantified following the Bradford (1976) assay, with slight modifications. Fresh leaf tissue (100 mg) was homogenized in 1 mL of ice-cold protein extraction buffer containing 10 mM Tris-HCl (pH 8.0), 1 mM EDTA, 0.1 mg mL^−1^ phenylmethylsulphonyl fluoride (PMSF), and 5 mM β-mercaptoethanol. Homogenization was carried out using a chilled mortar and pestle to prevent protein degradation. The homogenate was transferred to 1.5 mL microcentrifuge tubes and centrifuged at 12,000 rpm for 20 min at 4 °C. The resulting supernatant was collected and used for protein estimation. An aliquot of 100 μL of the supernatant was mixed with 3 mL of Bradford reagent, vortexed thoroughly, and incubated at room temperature for 10 min to allow color development. Absorbance was measured at 595 nm using a UV–visible spectrophotometer. A standard calibration curve was generated using bovine serum albumin (BSA) at concentrations ranging from 5 to 100 μg mL^−1^. Total soluble protein content in the samples was determined from the standard curve and expressed on a fresh weight basis.

### 2.11 Determination of soil pH and electrical conductivity (EC)

Soil pH and electrical conductivity (EC) were determined before sowing and after harvest (70 DAS). Before sowing, composite soil samples were collected from the prepared potting mixture, whereas after harvest, soil samples were collected separately from each treatment. The samples were air-dried at room temperature, gently crushed, and passed through a 2-mm sieve. Soil pH and EC were measured using a 1:2.5 (w/v) soil-to-deionized water suspension. The suspension was shaken for 30 min and allowed to settle before measurement. Soil pH was determined using a calibrated digital pH meter, and EC was measured using a calibrated conductivity meter at 25°C. Electrical conductivity values were expressed as dS m^−1^.

### 2.12 Soil metagenomic analysis

Rhizosphere soil samples were collected at harvest (70 DAS) from VBN8 and VBN11 plants under the control treatment (T1; without bacterial inoculation and NaCl) and consortium-inoculated plants subjected to high salinity stress (T6; 100 mM NaCl). The bacterial consortium was applied at 10, 25, and 45 DAS (2 mL pot^−1^). Total rhizosphere soil DNA was extracted using the Xploregen Soil DNA Extraction Kit according to the manufacturer’s instructions. DNA quality and purity were assessed using NanoDrop spectrophotometry and agarose gel electrophoresis. The V3–V4 hypervariable region of the bacterial 16S rRNA gene was amplified using the universal primers 16sF (5′-CCTACGGGNGGCWGCAG-3′) and 16sR (5′-GACTACHVGGGTATCTAATCC-3′). Sequencing libraries were prepared using Illumina barcoded adapters, purified with AMPure beads, and quantified using the Qubit dsDNA High Sensitivity Assay Kit. Paired-end sequencing (2 × 300 bp) was performed on the Illumina MiSeq platform.

Raw sequencing reads were quality-checked using FastQC (v0.11.9) and MultiQC (v1.10.1). Subsequent analyses, including read trimming, paired-end merging, chimera removal, operational taxonomic unit (OTU) clustering at 97% sequence similarity, representative sequence selection, and taxonomic assignment, were conducted using the Biokart metagenomics pipeline. Taxonomic annotation was performed against the NCBI reference database. Microbial community structure was evaluated using alpha diversity indices (Chao1, Shannon, Simpson, and Fisher) and beta diversity analysis through principal coordinate analysis (PCoA) based on Bray–Curtis dissimilarity. Rarefaction analysis, taxonomic profiling, relative abundance analysis, heatmap visualization, and core microbiome analysis were also performed to assess rhizosphere microbial community composition under different treatments.

### 2.13 Statistical analysis

Statistical analyses were performed using JMP Pro 18 software. Data are presented as mean ± standard error (SE) of five biological replicates (*n* = 5). A two-way analysis of variance (ANOVA) was conducted to evaluate the statistical significance of variety (V), treatments (T), and their interaction (V × T). When significant differences were observed, means were separated using Tukey’s honestly significant difference (HSD) test at *p* < 0.05.

## 3 Results

### 3.1 Compatibility assessment of HPGPB isolates

**FIGURE 1:**
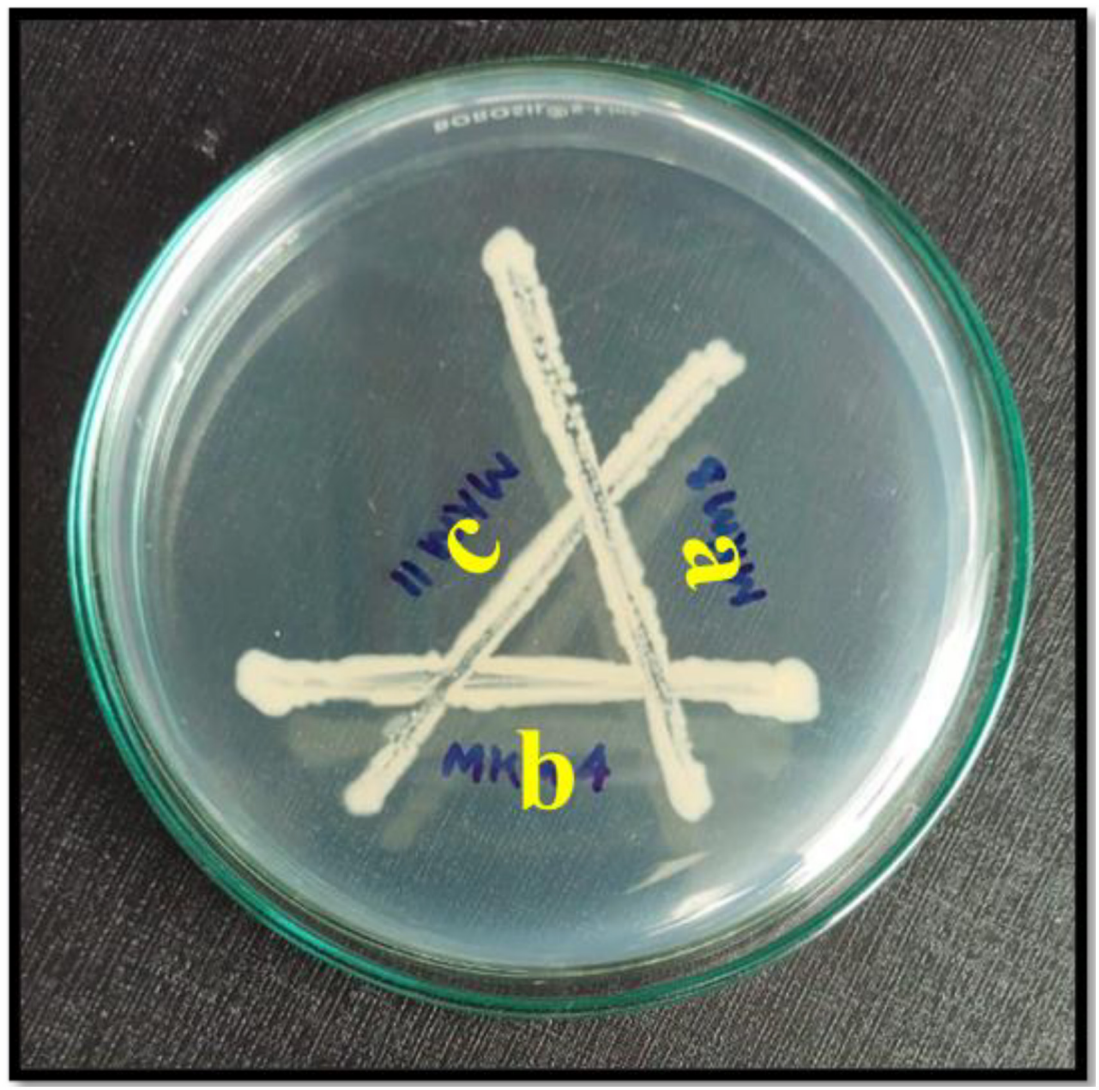
Compatibility assessment of bacterial isolates using the cross-streak plate method: **(a)** MKM3, **(b)** MKM4, and **(c)** MKM11.

The compatibility of the selected HPGPB isolates MKM3, MKM4, and MKM11 was evaluated using a cross-streak assay before consortium formulation. As shown in Figure 1, all paired combinations exhibited uniform and uninterrupted growth in the regions where the streaks approached or intersected. No visible zones of inhibition, growth suppression, or morphological abnormalities were observed among the tested isolates.

The absence of antagonistic interactions under the experimental conditions indicates that the isolates did not produce diffusible inhibitory compounds against one another on the halophilic agar medium. These observations were consistent across three independent replicates, confirming the stability and reproducibility of their interaction pattern. These findings confirmed the compatibility of MKM3, MKM4, and MKM11 for consortium formulation.

### 3.2 Greenhouse experiment

**FIGURE 2:**
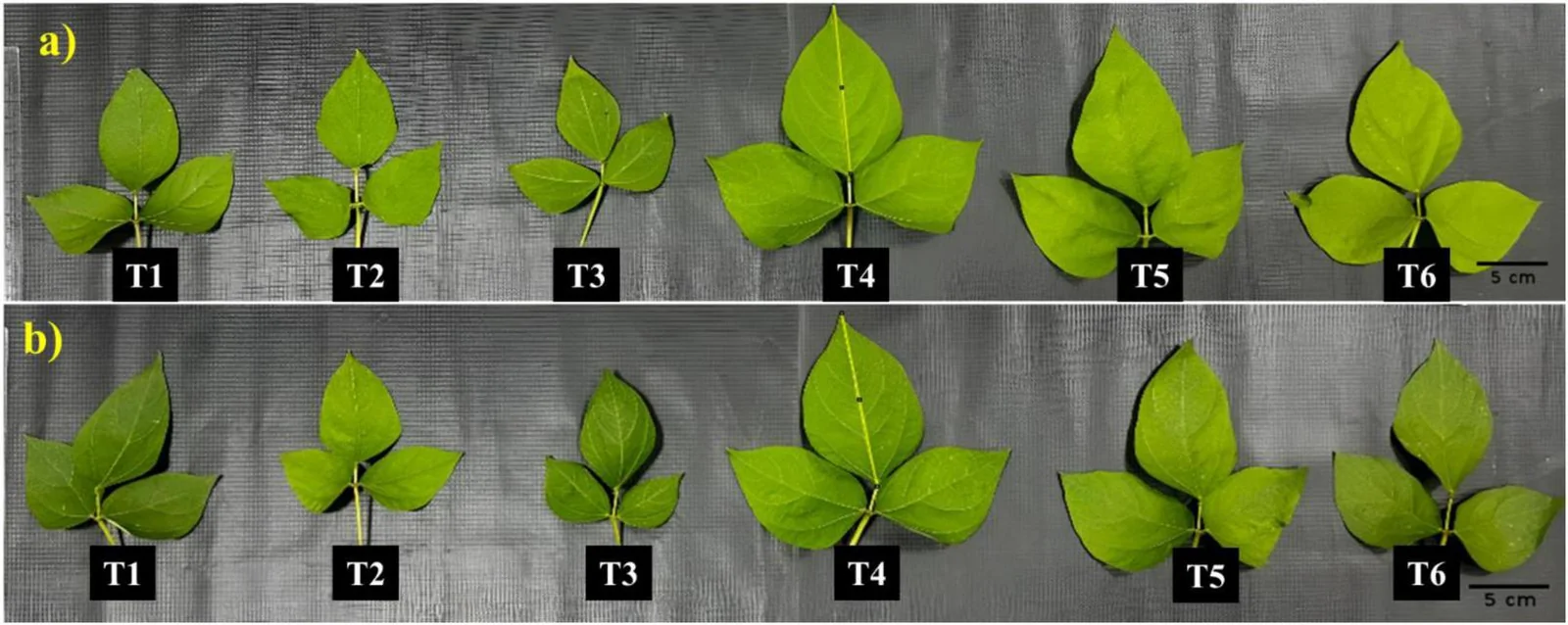
Leaf morphology of two black gram varieties: **(a)** VBN8, **(b)** VBN11, under different treatments T1, control without bacterial inoculation and NaCl; T2, plants exposed to 50 mM NaCl without bacterial inoculation; T3, plants exposed to 100 mM NaCl without bacterial inoculation; T4, plants inoculated with the bacterial consortium under non-saline conditions; T5, consortium-inoculated plants subjected to moderate salinity (50 mM NaCl); T6, consortium-inoculated plants subjected to high salinity stress (100 mM NaCl).

**FIGURE 3:**
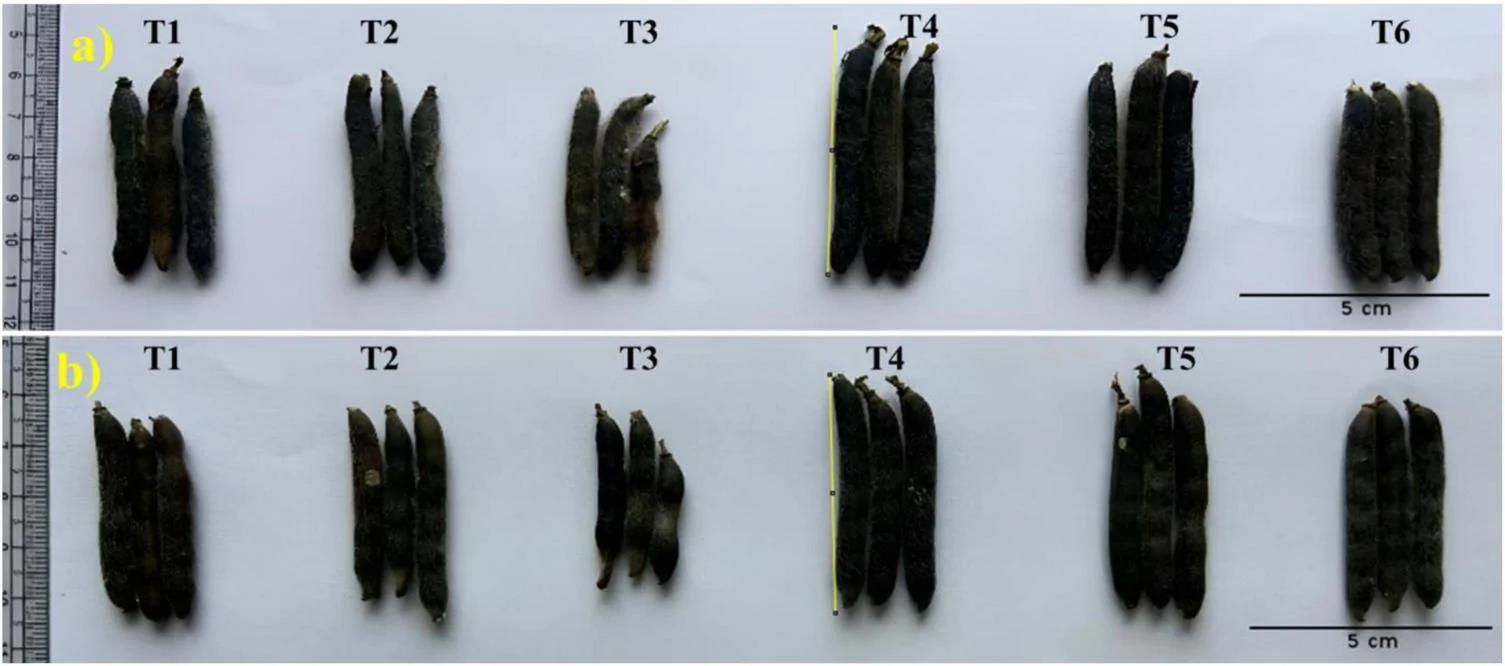
Pod morphology of two black gram varieties: **(a)** VBN8, **(b)** VBN11, under different treatments T1, control without bacterial inoculation and NaCl; T2, plants exposed to 50 mM NaCl without bacterial inoculation; T3, plants exposed to 100 mM NaCl without bacterial inoculation; T4, plants inoculated with the bacterial consortium under non-saline conditions; T5, consortium-inoculated plants subjected to moderate salinity (50 mM NaCl); T6, consortium-inoculated plants subjected to high salinity stress (100 mM NaCl).

A greenhouse experiment was conducted to evaluate the effects of an HPGPB consortium (MKM3, MKM4, and MKM11) on two black gram varieties (VBN8 and VBN11) under 50 and 100 mM NaCl stress (Supplementary file 1). Salt stress adversely affected morphological traits, including plant height, root length, nodulation, and leaf area, as well as biomass, grain yield, 100-grain weight, gas-exchange parameters, photosynthetic pigments, and total soluble protein (Figures 2, 3). In contrast, proline accumulation, malondialdehyde (MDA) content, and antioxidant enzyme activities increased under saline conditions, indicating enhanced osmotic and oxidative stress.

Consortium inoculation effectively mitigated the adverse effects of salinity. Inoculated plants showed significant improvements in growth, biomass, yield attributes, photosynthetic performance, pigment content, and protein levels compared with uninoculated salt-stressed plants. In addition, lipid peroxidation was reduced, while antioxidant enzyme activities were further enhanced, reflecting improved stress tolerance and redox balance. All observed effects were statistically significant (*p* < 0.05), confirming the efficacy of the bacterial consortium in alleviating salinity stress. Between the two varieties, VBN11 consistently exhibited superior performance under saline conditions and in response to consortium inoculation compared with VBN8.

### 3.3 Morphological attributes

**FIGURE 4:**
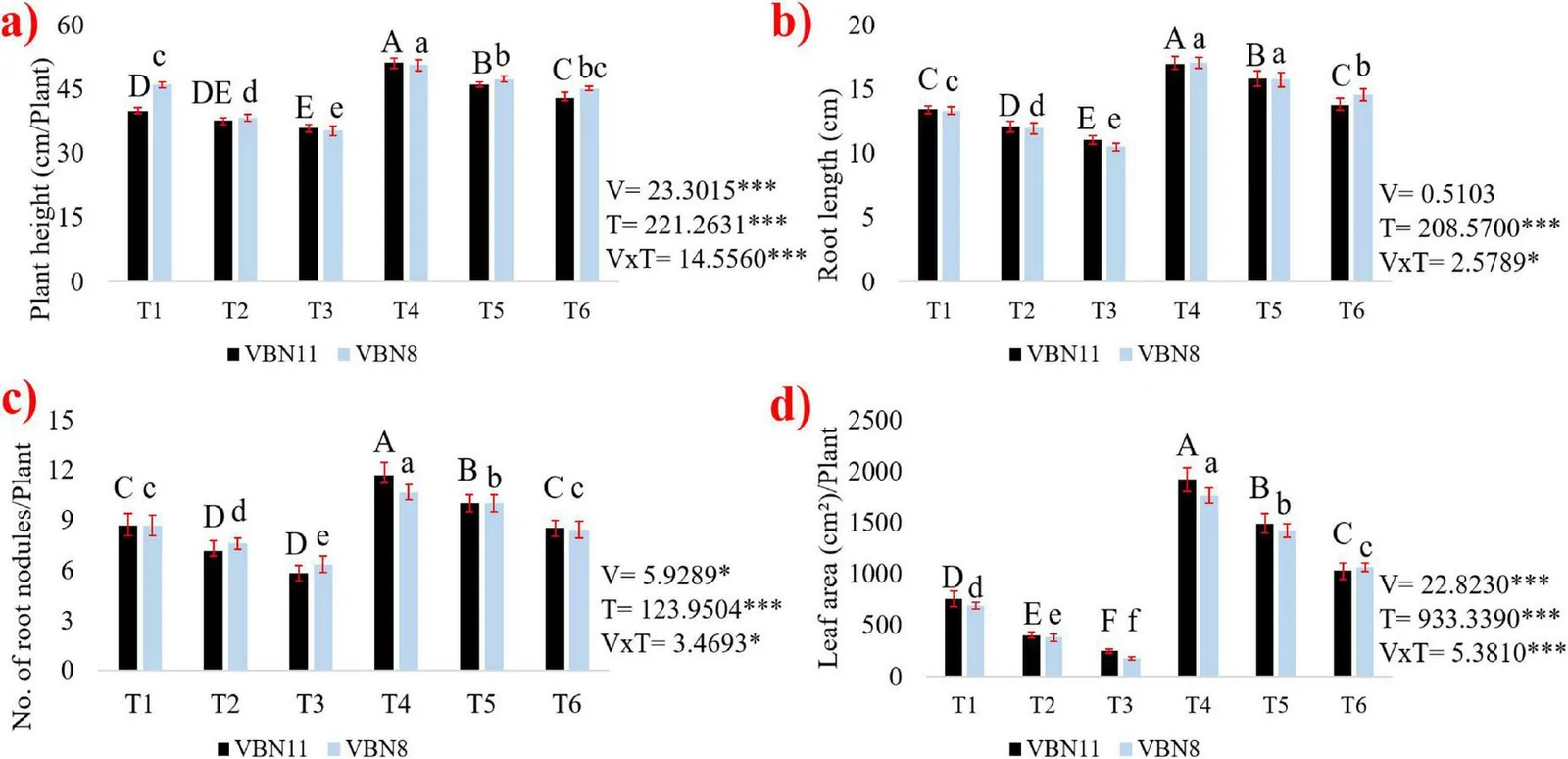
Morphological responses of black gram varieties VBN8 and VBN11 to HPGPB consortium inoculation under salinity stress. **(a)** Plant height, **(b)** root length, **(c)** leaf area, and **(d)** number of root nodules. Data are presented as mean ± SE (*n* = 5). Different letters indicate significant differences among treatments according to Tukey’s HSD test (*p* < 0.05). F-values for variety (V), treatment (T), and V × T interaction obtained from two-way ANOVA are shown within each panel. Treatment abbreviations: T1, control; T2, 50 mM NaCl; T3, 100 mM NaCl; T4, consortium inoculation; T5, consortium + 50 mM NaCl; and T6, consortium + 100 mM NaCl.

Salinity stress significantly reduced plant height, root length, number of root nodules, and leaf area in both black gram varieties (Figure 4). The magnitude of reduction increased with increasing salinity. In VBN11, moderate salinity (T2) decreased these traits by 4.6–43.5%, whereas severe salinity (T3) resulted in reductions of 12.8–68.6% relative to the control (T1). Similarly, VBN8 exhibited reductions of 5.8–45.3% under T2 and 12.7–71.1% under T3, indicating that the reductions became more pronounced with increasing salinity. Two-way ANOVA revealed significant treatment (T) effects for all morphological parameters (*p* < 0.001). Variety (V) significantly influenced plant height, number of root nodules, and leaf area (*p* < 0.001), whereas root length was not significantly affected by variety (*p* > 0.05). Significant V × T interactions were observed for plant height (*p* < 0.001), root length (*p* < 0.05), number of root nodules (*p* < 0.05), and leaf area (*p* < 0.001), indicating that the responses of the two varieties differed among treatments.

Consortium inoculation significantly improved all morphological traits under both non-saline and saline conditions. Under non-saline conditions (T4), inoculated plants exhibited greater plant height, root length, number of root nodules, and leaf area than the uninoculated control (T1). Similar improvements were observed under both moderate (T5 vs. T2) and severe (T6 vs. T3) salinity, demonstrating the effectiveness of the HPGPB consortium in mitigating the adverse effects of salt stress. Root nodulation showed the most pronounced response to consortium inoculation across all treatment conditions.

### 3.4 Physiological responses under salinity stress

**FIGURE 5:**
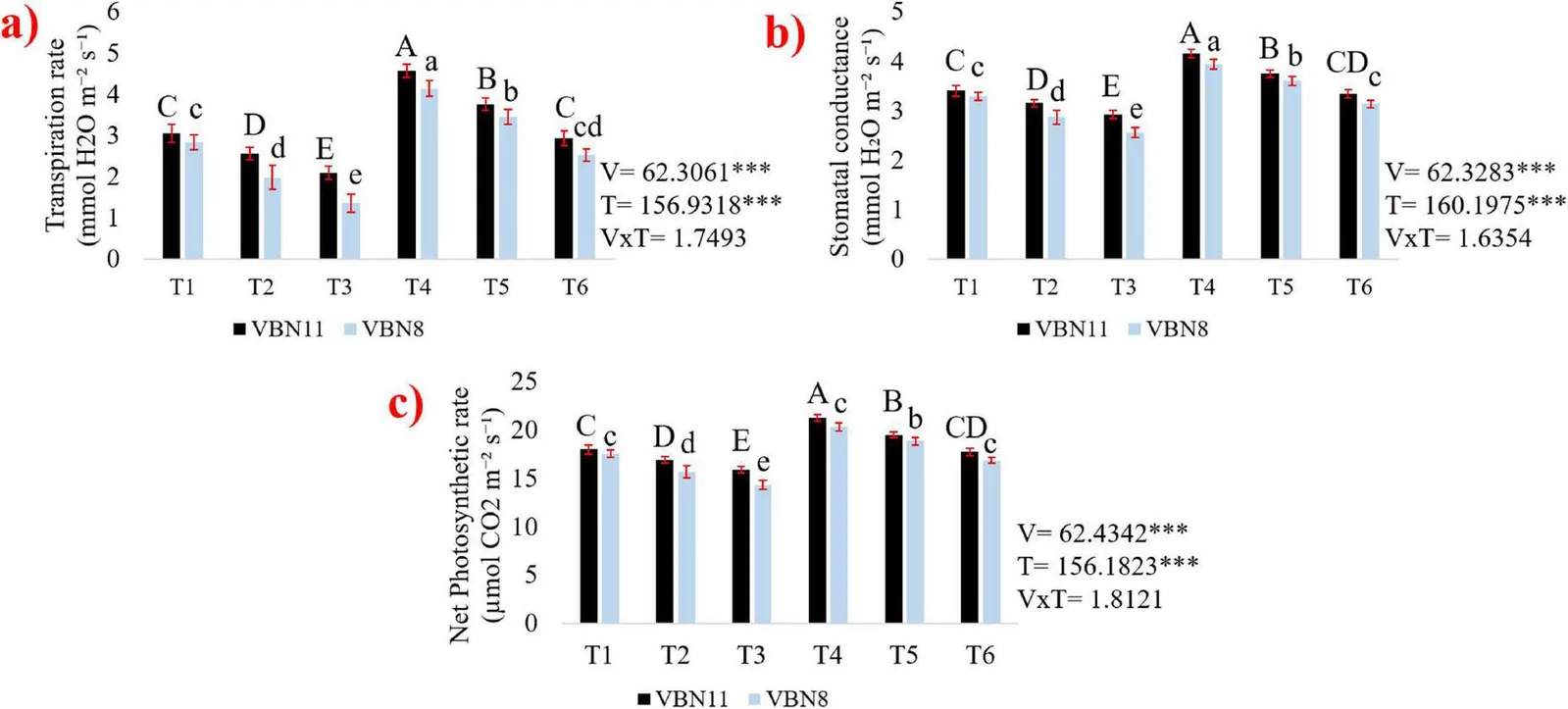
Physiological responses of black gram varieties VBN8 and VBN11 to HPGPB consortium inoculation under salinity stress. **(a)** Transpiration rate, **(b)** stomatal conductance, and **(c)** net photosynthetic rate. Data are presented as mean ± SE (*n* = 5). Different letters indicate significant differences among treatments according to Tukey’s HSD test (*p* < 0.05). F-values for variety (V), treatment (T), and V × T interaction obtained from two-way ANOVA are shown within each panel. Treatment abbreviations: T1, control; T2, 50 mM NaCl; T3, 100 mM NaCl; T4, consortium inoculation; T5, consortium + 50 mM NaCl; and T6, consortium + 100 mM NaCl.

Salinity stress significantly reduced transpiration rate, stomatal conductance, and net photosynthetic rate in both black gram varieties compared with the control (T1) (Figure 5). The reduction became more pronounced with increasing salinity. In VBN11, moderate salinity (T2) decreased these physiological parameters by 4.8–17.4%, whereas severe salinity (T3) caused reductions of 13.2–39.7% relative to the control. Similarly, VBN8 exhibited reductions of 5.9–16.1% under T2 and 12.5–37.3% under T3, indicating a progressive decline in physiological performance with increasing salinity. Two-way ANOVA revealed significant effects of variety (V) and treatment (T) for transpiration rate, stomatal conductance, and net photosynthetic rate (*p* < 0.001). However, the V × T interactions were not significant for transpiration rate (*F* = 1.75, *p* > 0.05), stomatal conductance (*F* = 1.64, *p* > 0.05), or net photosynthetic rate (*F* = 1.82, *p* > 0.05), indicating that both varieties exhibited similar physiological responses to salinity stress and consortium inoculation.

Consortium inoculation significantly improved all physiological parameters under both non-saline and saline conditions. Under non-saline conditions (T4), transpiration rate, stomatal conductance, and net photosynthetic rate increased by 10.4–42.1% in VBN11 and 13.3–46.0% in VBN8 compared with the control. Under moderate salinity (T5 vs. T2), these parameters increased by 10.1–49.2% in VBN11 and 12.5–48.1% in VBN8. Under severe salinity (T6 vs. T3), corresponding improvements ranged from 11.9 to 40.8% in VBN11 and 13.0–35.4% in VBN8. Overall, consortium inoculation effectively alleviated the salinity-induced decline in physiological performance in both black gram varieties.

### 3.5 Proline and lipid peroxidation under salinity stress

**FIGURE 6:**
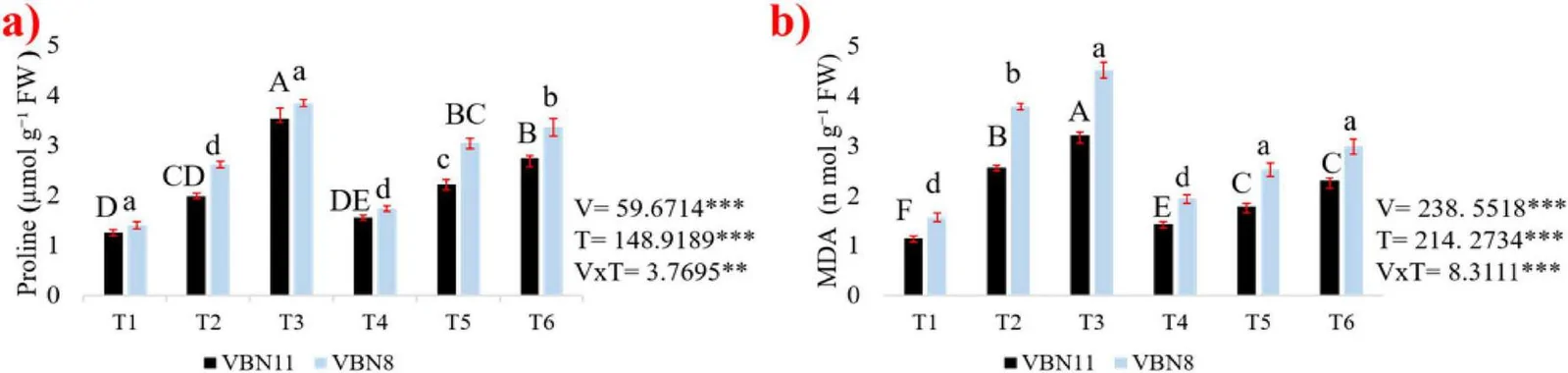
Changes in stress indicators of black gram varieties VBN8 and VBN11 in response to HPGPB consortium inoculation under salinity stress. **(a)** Proline content and **(b)** malondialdehyde (MDA) content. Data are presented as mean ± SE (*n* = 5). Different letters indicate significant differences among treatments according to Tukey’s HSD test (*p* < 0.05). *F*-values for variety (V), treatment (T), and V × T interaction obtained from two-way ANOVA are shown within each panel. Treatment abbreviations: T1, control; T2, 50 mM NaCl; T3, 100 mM NaCl; T4, consortium inoculation; T5, consortium + 50 mM NaCl; and T6, consortium + 100 mM NaCl.

Salinity stress significantly increased proline and malondialdehyde (MDA) contents in both black gram varieties compared with the control (T1) (Figure 6). The magnitude of increase was greater under severe salinity than under moderate salinity. In VBN11, moderate salinity (T2) increased proline and MDA contents by 88.4 and 132.5%, respectively, whereas severe salinity (T3) resulted in increases of 175.2 and 170.5% relative to the control. Similarly, in VBN8, proline content increased by 56.6 under T2 and 170.9% under T3, while MDA content increased by 114.4 and 278.2%, respectively. Two-way ANOVA revealed significant effects of variety (V), treatment (T), and the V × T interaction on both proline and MDA contents (*p* < 0.001). The significant V × T interactions for proline (*F* = 3.77, *p* < 0.001) and MDA (*F* = 8.31, *p* < 0.001) indicate that the magnitude of the salinity response and the effect of consortium inoculation differed between VBN11 and VBN8.

Consortium inoculation significantly modulated both stress indicators under saline conditions. Under moderate salinity (T5 vs. T2), proline content increased by 18.5% in VBN11 and 12.3% in VBN8, whereas MDA content decreased by 28.2 and 28.0%, respectively. Under severe salinity (T6 vs. T3), proline content decreased by 11.4 in VBN11 and 12.4% in VBN8, while MDA content declined by 30.4 and 33.5%, respectively. Overall, consortium inoculation reduced lipid peroxidation under both salinity levels, while proline accumulation was differentially regulated depending on the severity of salinity stress.

### 3.6 Antioxidant enzyme activity under salinity stress

**FIGURE 7:**
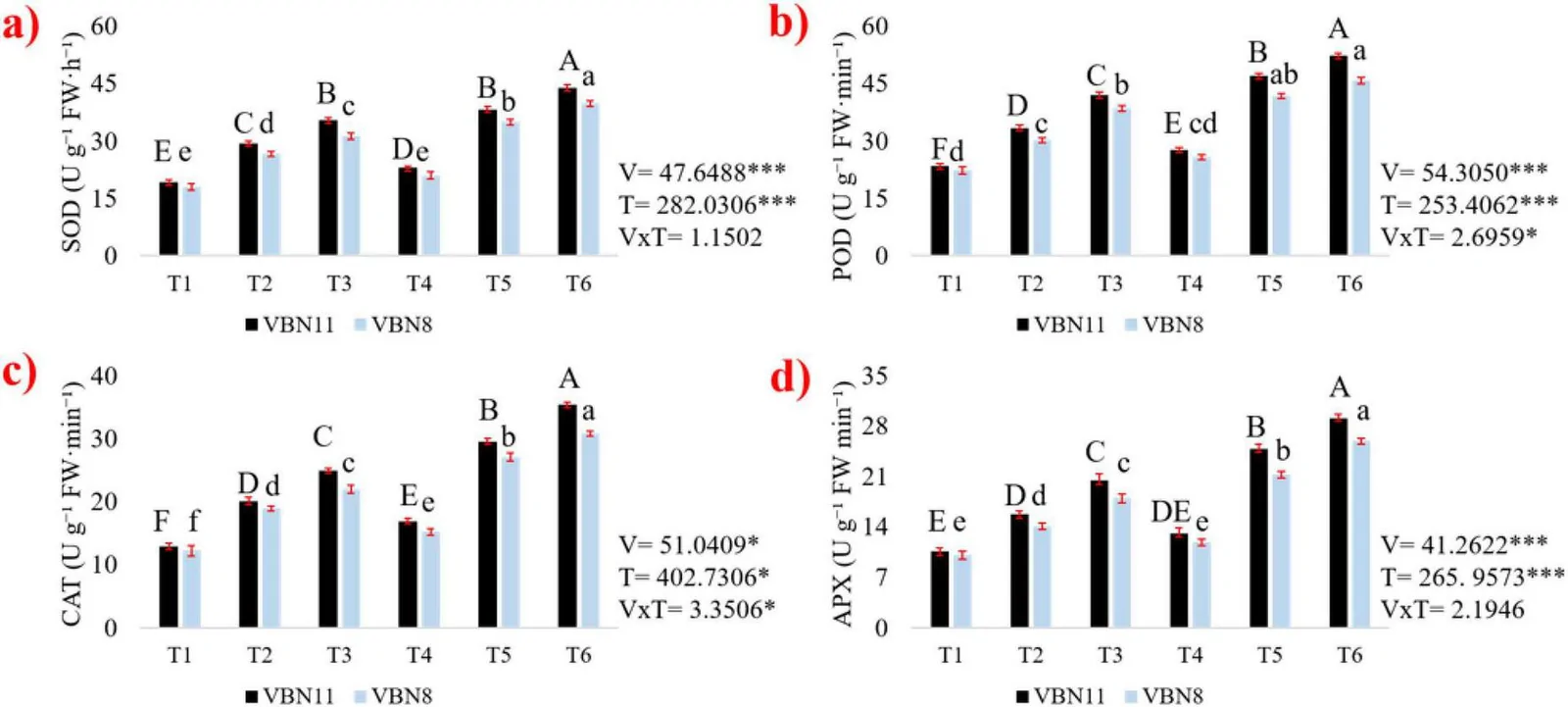
Antioxidant enzyme activities in black gram varieties VBN8 and VBN11 in response to HPGPB consortium inoculation under salinity stress. **(a)** Superoxide dismutase (SOD), **(b)** peroxidase (POD), **(c)** catalase (CAT), and **(d)** ascorbate peroxidase (APX). Data are presented as mean ± SE (*n* = 5). Different letters indicate significant differences among treatments according to Tukey’s HSD test (*p* < 0.05). *F*-values for variety (V), treatment (T), and V × T interaction obtained from two-way ANOVA are shown within each panel. Treatment abbreviations: T1, control; T2, 50 mM NaCl; T3, 100 mM NaCl; T4, consortium inoculation; T5, consortium ++ 50 mM NaCl; and T6, consortium ++ 100 mM NaCl.

Salinity stress significantly increased the activities of superoxide dismutase (SOD), peroxidase (POD), catalase (CAT), and ascorbate peroxidase (APX) in both black gram varieties compared with the control (T1) (Figure 7). The magnitude of enzyme activity increased with salinity level. In VBN11, moderate salinity (T2) increased SOD, POD, CAT, and APX activities by 47.9, 27.5, 59.7, and 41.8%, respectively, whereas severe salinity (T3) resulted in increases of 79.3, 71.5, 93.8, and 67.7%, respectively. Similarly, in VBN8, moderate salinity increased SOD, POD, CAT, and APX activities by 57.5, 37.4, 53.7, and 50.7%, respectively, while severe salinity resulted in increases of 89.0, 72.0, 87.3, and 91.5%, respectively. Two-way ANOVA revealed significant effects of variety (V) and treatment (T) for all antioxidant enzymes (*p* < 0.001). Significant V × T interactions were observed for POD (*F* = 2.70, *p* < 0.05) and CAT (*F* = 3.35, *p* < 0.01), indicating that the magnitude of treatment responses differed between VBN11 and VBN8. In contrast, the V × T interactions were not significant for SOD (*F* = 1.15, *p* > 0.05) or APX (*F* = 2.19, *p* > 0.05), indicating similar responses of both varieties for these enzymes.

Consortium inoculation further enhanced antioxidant enzyme activities under both moderate and severe salinity conditions. Under moderate salinity (T5 vs. T2), SOD, POD, CAT, and APX activities increased by 34.9, 39.5, 45.5, and 43.6%, respectively, in VBN11, and by 27.7, 43.8, 45.8, and 60.5%, respectively, in VBN8. Under severe salinity (T6 vs. T3), the corresponding increases were 23.4, 18.8, 34.6, and 43.0% in VBN11, and 27.2, 25.0, 44.0, and 37.5% in VBN8. Overall, consortium inoculation consistently enhanced antioxidant enzyme activities under both salinity levels in both black gram varieties.

### 3.7 Photosynthetic pigments under salinity stress

**FIGURE 8:**
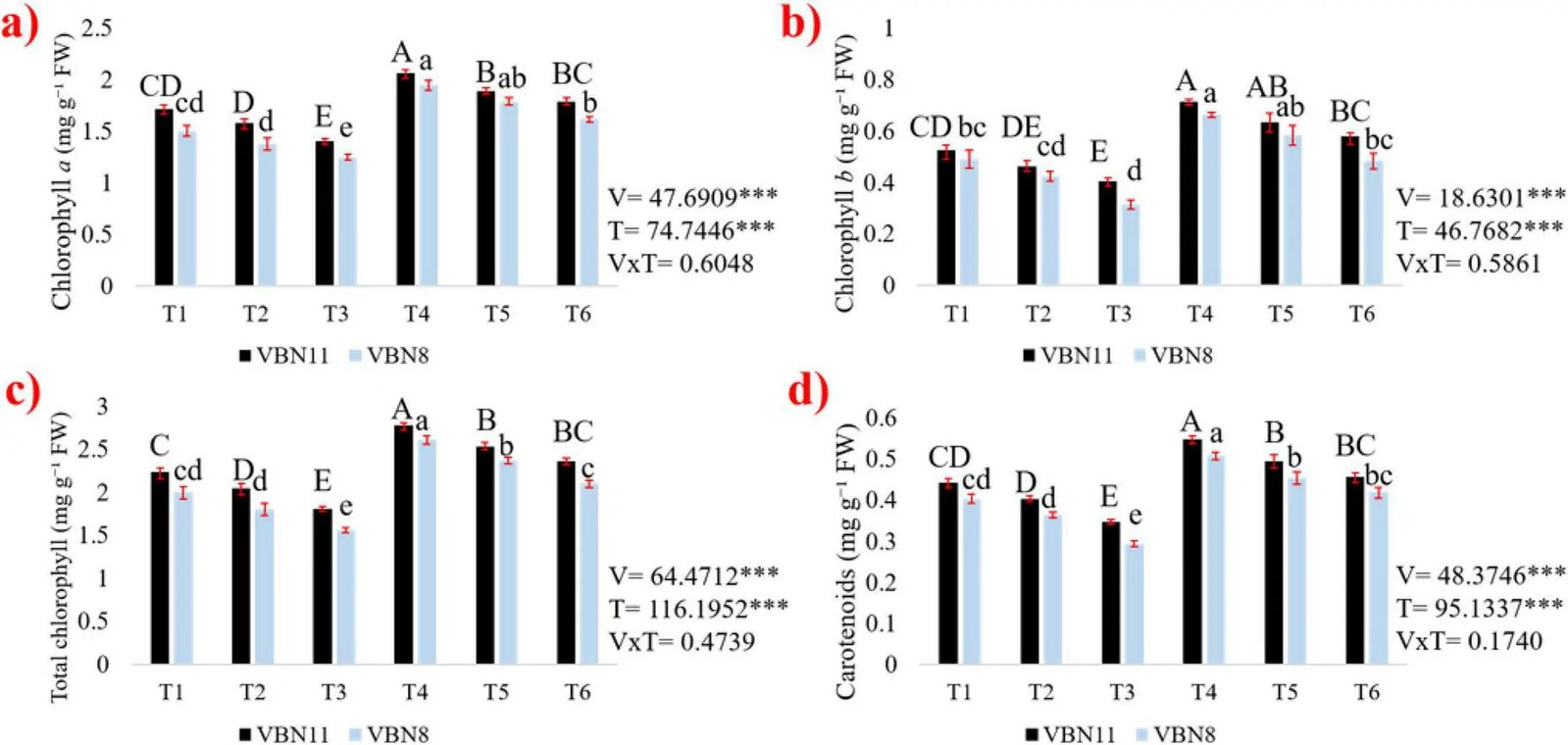
Photosynthetic pigment contents of black gram varieties VBN8 and VBN11 in response to HPGPB consortium inoculation under salinity stress. **(a)** Chlorophyll a, **(b)** chlorophyll b, **(c)** total chlorophyll, and **(d)** carotenoids. Data are presented as mean ± SE (*n* = 5). Different letters indicate significant differences among treatments according to Tukey’s HSD test (*p* < 0.05). *F*-values for variety (V), treatment (T), and V × T interaction obtained from two-way ANOVA are shown within each panel. Treatment abbreviations: T1, control; T2, 50 mM NaCl; T3, 100 mM NaCl; T4, consortium inoculation; T5, consortium + 50 mM NaCl; and T6, consortium + 100 mM NaCl.

Salinity stress significantly reduced chlorophyll a (Chl *a*), chlorophyll *b* (Chl *b*), total chlorophyll (Chl total), and carotenoid contents in both black gram varieties compared with the control (T1) (Figure 8). The reduction in pigment content became more pronounced with increasing salinity. In VBN8, moderate salinity (T2) decreased photosynthetic pigments by 8.6–14.0%, whereas severe salinity (T3) resulted in greater reductions of 17.7–37.3% relative to the control. Similarly, in VBN11, pigment contents declined by 7.5–10.6% under T2 and by 19.4–22.9% under T3. Two-way ANOVA revealed significant effects of variety (V) and treatment (T) for all photosynthetic pigments (*p* < 0.001). However, the V × T interactions were not significant for chlorophyll a (*F* = 0.60, *p* > 0.05), chlorophyll b (*F* = 0.59, *p* > 0.05), total chlorophyll (*F* = 0.47, *p* > 0.05), or carotenoids (*F* = 0.17, *p* > 0.05), indicating that both varieties exhibited similar responses to salinity stress and consortium inoculation.

Consortium inoculation significantly increased photosynthetic pigment contents under both non-saline and saline conditions. Under non-saline conditions (T4), chlorophyll a, chlorophyll b, total chlorophyll, and carotenoid contents increased by 28.6–51.6% in VBN8 and 21.2–35.6% in VBN11 compared with the control. Under moderate salinity (T5 vs. T2), pigment contents increased by 27.8–42.3% in VBN8 and 18.9–35.2% in VBN11. Under severe salinity (T6 vs. T3), the corresponding increases ranged from 25.8 to 62.1% in VBN8 and 28.6–46.7% in VBN11. Overall, consortium inoculation effectively mitigated the salinity-induced decline in photosynthetic pigment contents in both black gram varieties.

### 3.8 Yield Attributes under salinity stress

**FIGURE 9:**
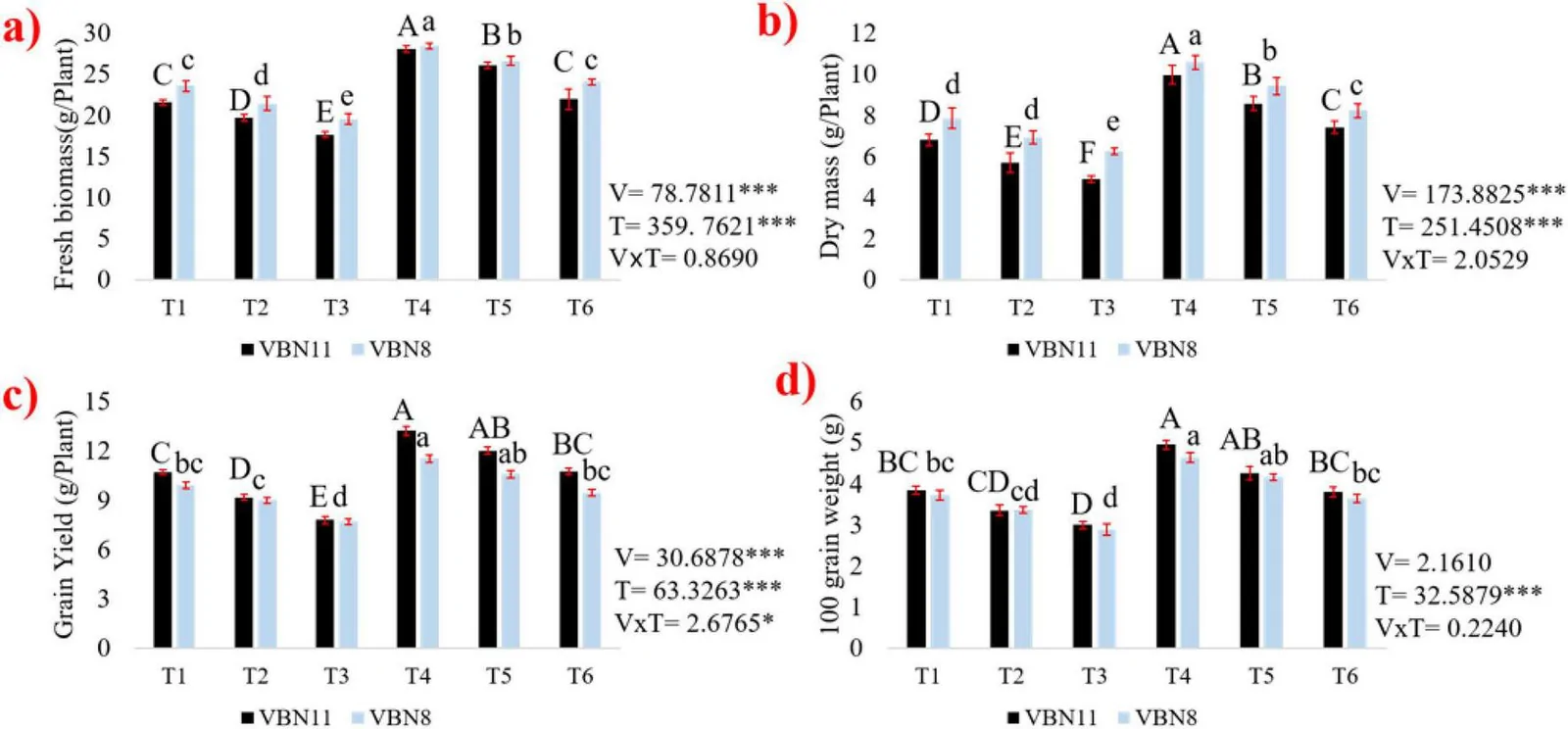
Yield attributes of black gram varieties VBN8 and VBN11 in response to HPGPB consortium inoculation under salinity stress. **(a)** Fresh biomass, **(b)** dry biomass, **(c)** grain yield, and **(d)** 100-grain weight. Data are presented as mean ± SE (*n* = 5). Different letters indicate significant differences among treatments according to Tukey’s HSD test (*p* < 0.05). *F*-values for variety (V), treatment (T), and V × T interaction obtained from two-way ANOVA are shown within each panel. Treatment abbreviations: T1, control; T2, 50 mM NaCl; T3, 100 mM NaCl; T4, consortium inoculation; T5, consortium + 50 mM NaCl; and T6, consortium + 100 mM NaCl.

Salinity stress significantly reduced fresh biomass, dry biomass, grain yield, and 100-grain weight in both black gram varieties compared with the control (T1) (Figure 9). The reduction in yield attributes became more pronounced with increasing salinity. In VBN11, moderate salinity (T2) reduced these traits by 11.8–18.5%, whereas severe salinity (T3) resulted in reductions of 21.9–30.7% relative to the control. Similarly, in VBN8, fresh biomass, dry biomass, grain yield, and 100-grain weight declined by 9.2–17.7% under T2 and by 21.3–31.4% under T3. Two-way ANOVA revealed significant treatment (T) effects for all yield attributes (*p* < 0.001). Variety (V) significantly affected fresh biomass, dry biomass, and grain yield (*p* < 0.001), whereas 100-grain weight was not significantly influenced by variety (*p* > 0.05). A significant V × T interaction was observed only for grain yield (*F* = 2.68, *p* < 0.05), indicating that the response of grain yield to salinity stress and consortium inoculation differed between VBN11 and VBN8. In contrast, the V × T interactions were not significant for fresh biomass (*F* = 0.87, *p* > 0.05), dry biomass (*F* = 2.05, *p* > 0.05), or 100-grain weight (*F* = 0.22, *p* > 0.05), indicating similar treatment responses in both varieties for these traits.

Consortium inoculation significantly improved all yield attributes under both non-saline and saline conditions. Under non-saline conditions (T4), fresh biomass, dry biomass, grain yield, and 100-grain weight increased by 24.9–40.8% in VBN11 and 19.4–39.4% in VBN8 compared with the control. Under moderate salinity (T5 vs. T2), these traits increased by 20.8–46.2% in VBN11 and 24.5–32.3% in VBN8. Under severe salinity (T6 vs. T3), the corresponding increases ranged from 27.0 to 38.0% in VBN11 and 23.5–30.7% in VBN8. Overall, consortium inoculation effectively mitigated the adverse effects of salinity on biomass production and yield in both black gram varieties.

### 3.9 Total soluble protein

**FIGURE 10:**
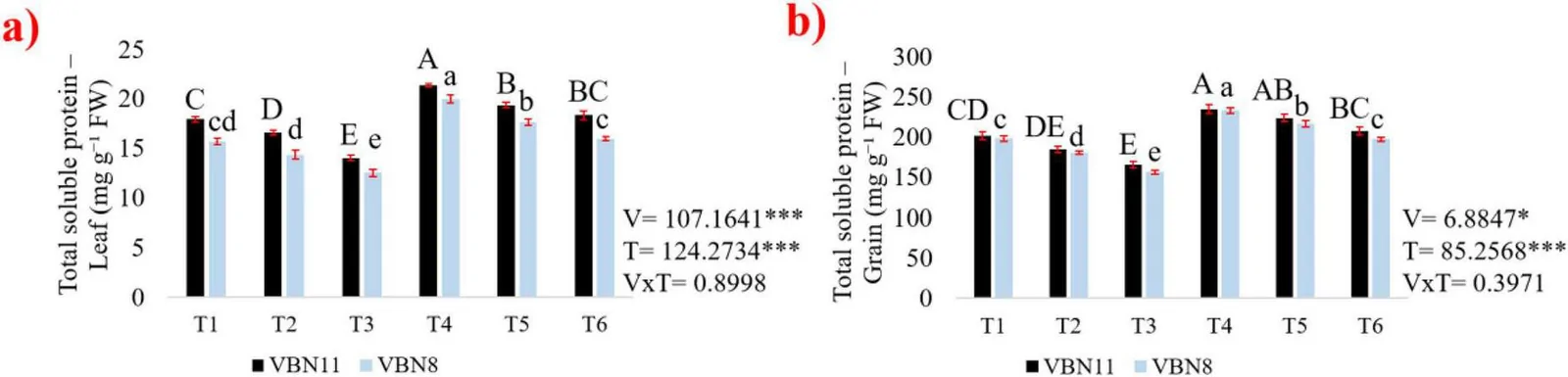
Protein content of black gram varieties VBN8 and VBN11 in response to HPGPB consortium inoculation under salinity stress. **(a)** Total soluble protein in leaves and **(b)** total soluble protein in grains. Data are presented as mean ± SE (*n* = 5). Different letters indicate significant differences among treatments according to Tukey’s HSD test (*p* < 0.05). *F*-values for variety (V), treatment (T), and V × T interaction obtained from two-way ANOVA are shown within each panel. Treatment abbreviations: T1, control; T2, 50 mM NaCl; T3, 100 mM NaCl; T4, consortium inoculation; T5, consortium + 50 mM NaCl; and T6, consortium + 100 mM NaCl.

Salinity stress significantly reduced total soluble protein content in both leaves (TSPL) and grains (TSPG) of the two black gram varieties compared with the control (T1) (Figure 10). The reduction in protein content became more pronounced with increasing salinity. In VBN11, moderate salinity (T2) decreased TSPL and TSPG by 8.1 and 8.3%, respectively, whereas severe salinity (T3) resulted in reductions of 21.7 and 21.5% relative to the control. Similarly, in VBN8, TSPL and TSPG decreased by 5.8 and 8.7%, respectively, under T2 and by 17.5 and 20.8%, respectively, under T3. Two-way ANOVA revealed significant treatment (T) effects for both TSPL and TSPG (*p* < 0.001). Variety (V) also had a significant effect on TSPL (*F* = 107.16, *p* < 0.001) and TSPG (*F* = 6.88, *p* < 0.05). However, the V × T interactions were not significant for either TSPL (*F* = 0.90, *p* > 0.05) or TSPG (*F* = 0.40, *p* > 0.05), indicating that both varieties exhibited similar responses to salinity stress and consortium inoculation.

Consortium inoculation significantly increased total soluble protein content under both non-saline and saline conditions. Under non-saline conditions (T4), TSPL and TSPG increased by 27.1 and 20.4%, respectively, in VBN11 and by 19.4 and 17.6%, respectively, in VBN8 compared with the control. Under moderate salinity (T5 vs. T2), TSPL increased by 20.8% in VBN11 and 15.5% in VBN8, whereas TSPG increased by 21.9 and 20.8%, respectively. Under severe salinity (T6 vs. T3), TSPL increased by 30.0% in VBN11 and 30.8% in VBN8, while TSPG increased by 29.0 and 26.0%, respectively. Overall, consortium inoculation effectively alleviated the salinity-induced reduction in total soluble protein content in both leaves and grains of the two black gram varieties.

### 3.10 Plant nutrient composition

The concentrations of macro- and micronutrients (N, P, K, Ca, Mg, Zn, and Fe) and Na were determined in plant tissues to evaluate nutrient acquisition and ionic homeostasis under salinity stress (Table 2). Significant variations were observed among treatments (T1–T6) in both VBN8 and VBN11. Salinity stress (T2 and T3) adversely affected nutrient accumulation, with T3 (severe salinity) recording the lowest concentrations of most macro- and micronutrients in both varieties. In particular, significant reductions in N, K, Mg, Zn, and Fe concentrations were observed under severe salinity, indicating impaired nutrient uptake and nutrient imbalance caused by salt stress. Conversely, Na^+^ concentration increased under salinity treatments, reflecting enhanced ionic toxicity and disruption of ion homeostasis. Consortium inoculation under non-saline conditions (T4) significantly enhanced nutrient accumulation. Both varieties recorded the highest concentrations of essential nutrients under T4, including nitrogen (2.34–2.41%), phosphorus (3.55%), potassium (2.23–2.33%), calcium (1.85–1.87%), zinc (34.80 mg kg^−1^), and iron (750.43 mg kg^−1^), demonstrating improved nutrient acquisition and assimilation.

**TABLE 2:** Characterization of plant growth-promoting traits and salt tolerance of selected HPGPB isolates.

Plant growth-promoting traits	MKM3	MKM4	MKM11
Ammonia production	–	–	+
IAA production	+	+	+
Phosphate solubilization	+	–	+
Potassium solubilization	+	+	–
Siderophore production	+	–	+
Oxidase activity	+	+	+
Catalase activity	+	+	+
Salt tolerance	Up to 15% NaCl	Up to 15% NaCl	Up to 20% NaCl

(+) Positive; (–) Negative.

Under saline conditions, consortium application (T5 and T6) substantially improved nutrient status compared with the uninoculated salinity treatments (T2 and T3). Higher concentrations of N, P, K, Ca, Mg, Zn, and Fe were observed in inoculated plants, indicating enhanced nutrient uptake under salt stress. Notably, consortium-treated plants exhibited reduced Na^+^ accumulation and increased K^+^ and Ca^2+^ concentrations compared with uninoculated saline treatments. The resulting improvement in the K^+^/Na^+^ balance suggests that the bacterial consortium was associated with improved ionic homeostasis under salinity stress. Overall, the nutrient composition data clearly demonstrate that salinity stress impaired nutrient acquisition and increased Na^+^ accumulation, whereas inoculation with the HPGPB consortium improved macro- and micronutrient uptake, reduced Na^+^ accumulation, and enhanced ionic balance in both black gram varieties (Table 3). Soil pH and electrical conductivity (EC) before sowing and after harvest are presented in Table 4.

**TABLE 3:** Influence of salinity stress and microbial consortium application on macro- and micronutrient concentrations in two black gram (*Vigna mungo* L.) varieties (VBN8 and VBN11).

Variety	Treatment	N (%)	P (%)	K (%)	Na (%)	Mg (%)	Ca (%)	Zn (mg kg^−1^)	Fe (mg kg^−1^)
VBN8	T1	1.807 ± 0.019 c	2.380 ± 0.047 bc	1.757 ± 0.050 cd	0.467 ± 0.009 e	0.383 ± 0.009 b	0.890± 0.017 c	31.563 ± 0.264 bc	587.727 ± 6.865 b
	T2	1.593 ± 0.018 d	1.507 ± 0.037 cd	1.630 ± 0.044 de	0.940 ±0.017 b	0.347 ± 0.024 b	0.817 ± 0.050 cd	29.357 ± 0.803 c	532.990 ± 5.214 c
	T3	1.493 ±0.045 d	1.317 ± 0.049 d	1.503 ± 0.055 e	1.050 ± ± 0.021 a	0.250 ± 0.017 c	0.717 ± 0.024 d	23.980 ± 0.560 d	514.523 ± 4.990 c
	T4	2.340 ± 0.051 a	3.550 ± 0.110 a	2.230 ± 0.053 a	0.580 ± 0.012 d	0.570 ± 0.010 a	1.850 ± 0.044 a	34.800 ± 0.902 a	750.433 ± 13.550 a
	T5	2.077 ± 0.024 b	3.133 ± 0.442 ab	2.067 ± 0.047 ab	0.700 ± 0.015 c	0.550 ± 0.015 a	1.293 ± 0.027 b	32.447 ±0.659 ab	624.867 ± 11.196 b
	T6	1.973 ± 0.074 bc	2.420 ± 0.076 b	1.867 ± 0.029 bc	0.910 ± 0.021 b	0.410 ± 0.012 b	1.230 ± 0.021 b	31.680 ± 0.426 bc	620.200 ± 10.955 b
VBN11	T1	1.873 ± 0.032 bc	2.447 ± 0.032 b	1.790 ± 0.032 bc	0.517 ± 0.032 d	0.397 ± 0.018 b	0.903 ± 0.019 c	31.633 ± 0.284 b	581.327 ± 7.133 bc
	T2	1.660 ± 0.035 cd	1.440 ± 0.031 c	1.677 ± 0.038 cd	0.977 ± 0.015 b	0.417 ± 0.032 b	0.843 ± 0.037 cd	30.217 ± 0.369 b	546.323 ± 7.653 cd
	T3	1.560 ± 0.072 d	1.327 ± 0.053 c	1.517 ± 0.054 d	1.157 ± 0.020 a	0.283 ± 0.009 c	0.757 ± 0.007 d	24.313 ± 0.498 c	525.523 ±5.369 d
	T4	2.407 ± 0.052 a	3.550 ± 0.110 a	2.330 ± 0.046 a	0.617 ± 0.022 cd	0.593 ± 0.030 a	1.873 ± 0.042 a	34.800 ± 0.902 a	750.433 ± 13.550 a
	T5	2.143 ± 0.057 ab	3.133 ± 0.442 ab	2.167 ± 0.023 a	0.727 ± 0.043 c	0.573 ± 0.009 a	1.317 ± 0.028 b	32.447 ± 0.659 ab	624.867 ± 11.196 b
	T6	1.940 ± 0.092 bc	2.420 ± 0.076 b	1.900 ± 0.042 b	0.927 ± 0.022 b	0.427 ± 0.020 b	1.250 ± 0.015 b	31.680 ± 0.426 b	620.200 ± 10.955 b

Data are presented as mean ± standard error (SE) of five biological replicates (*n* = 5). Different lowercase letters within a column indicate significant differences among treatments according to Tukey’s HSD test at *p* < 0.05.

**TABLE 4:** Comparative soil analysis (pH and EC) of VBN8 and VBN11 black gram varieties.

Parameter		VBN8		VBN11	
Overall soil analysis (before sowing)					
pH		7.6		7.6	
EC (dS m^−1^)		1.2		1.2	
**Treatment**	**Description**	**pH (VBN8)**	**pH (VBN11)**	**EC (VBN8) (dS m^−1^)**	**EC (VBN11) (dS m^−1^)**
Treatment-wise soil analysis (after harvest)					
T1	Control without bacterial inoculation and NaCl	6.8	6.9	1.0	1.0
T2	Plants exposed to 50 mM NaCl without bacterial inoculation	7.2	7.3	3.6	3.8
T3	Plants exposed to 100 mM NaCl without bacterial inoculation	7.4	7.5	6.6	6.9
T4	Plants inoculated with the bacterial consortium under non-saline conditions	6.7	6.8	0.6	0.7
T5	Consortium-inoculated plants subjected to moderate salinity (50 mM NaCl)	7.0	7.1	3.1	3.2
T6	Consortium-inoculated plants subjected to high salinity stress (100 mM NaCl).	7.2	7.3	5.7	6.1

### 3.11 Soil microbial community structure

Metagenomic analysis revealed a highly diverse microbial community in both VBN8 and VBN11 soils, with no single dominant taxon across treatments. However, consortium-treated soils (T6) showed noticeable shifts in microbial composition compared to control (T1), as indicated by differences in relative abundance profiles.

In T6 soils, a relatively higher abundance of halotolerant and plant growth-promoting genera such as *Bacillus*, *Pseudomonas*, *Halomonas*, and *Marinobacter* was observed compared to T1. Although halophilic-associated taxa were detected, they were present at low abundance and belonged to genera other than the inoculated *Halobacillus*. The inoculated strains were not detected at significant levels in the metagenomic dataset at 70 DAS (Supplementary file 2).

### 3.12 Phylum-level microbial community

**FIGURE 11:**
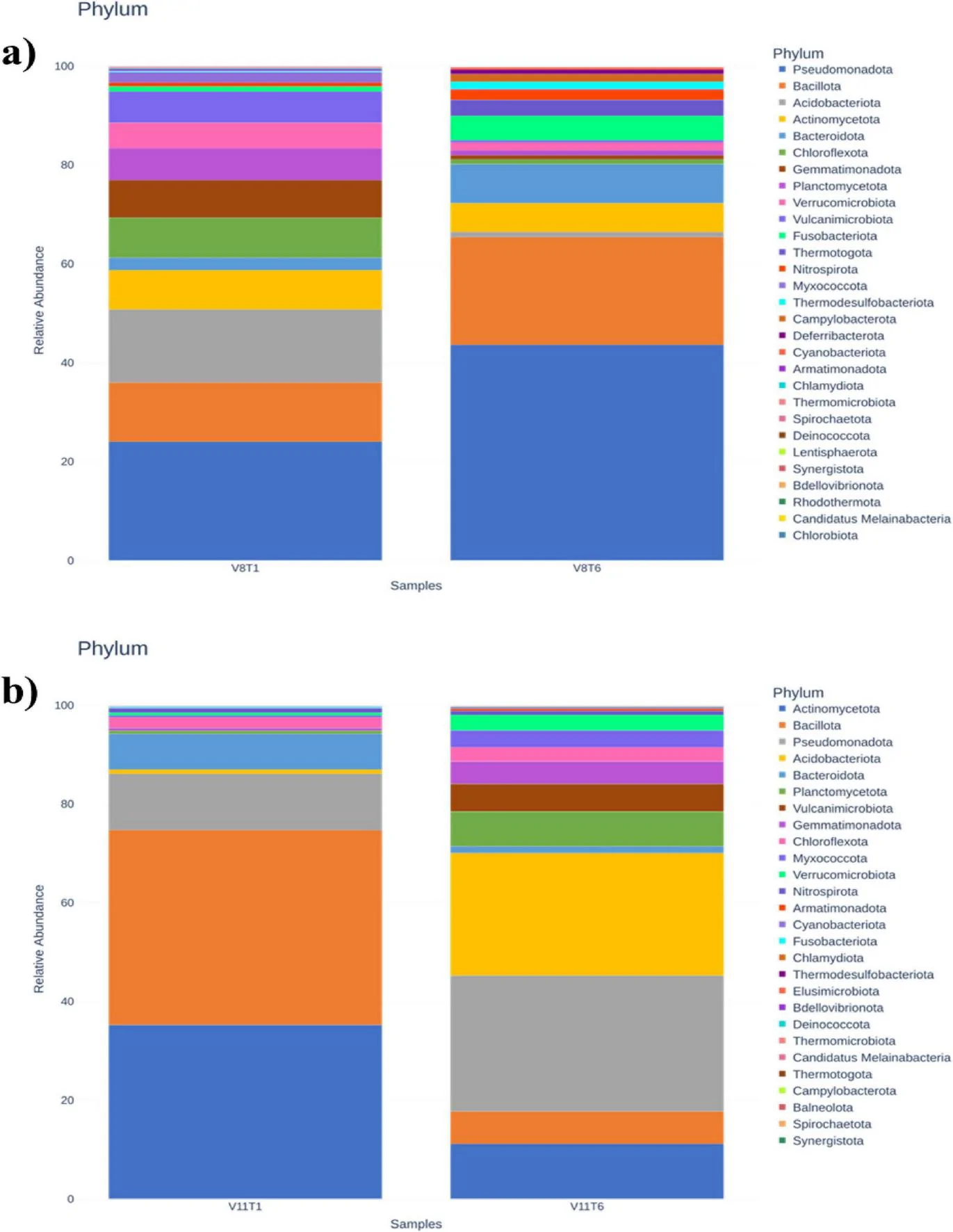
Phylum-level microbial community composition in VBN8 and VBN11 rhizosphere soils. Stacked bar plots showing the relative abundance (%) of dominant bacterial phyla in control (T1) and consortium-treated (T6) soils at harvest (70 DAS). **(a)** VBN8 and **(b)** VBN11. Consortium treatment altered the relative abundance of major phyla, including Proteobacteria, Firmicutes, Actinobacteria, and Bacteroidetes, indicating shifts in microbial community structure under saline conditions.

Phylum-level analysis showed that *Proteobacteria*, *Firmicutes*, *Actinomycetota*, and *Bacillota* dominated the rhizosphere communities of both VBN8 and VBN11 (Figure 11). In VBN8, consortium treatment (T6) increased the relative abundance of *Proteobacteria* and *Bacteroidota*, while *Actinomycetota* slightly decreased and Firmicutes remained stable. In VBN11, *Proteobacteria* decreased under T6, whereas *Actinomycetota* and *Bacteroidota* were enriched compared to the control (T1). Minor phyla showed low abundance and slight variation across treatments, indicating overall shifts in microbial composition without dominance by a single phylum.

### 3.13 Alpha diversity

**FIGURE 12:**
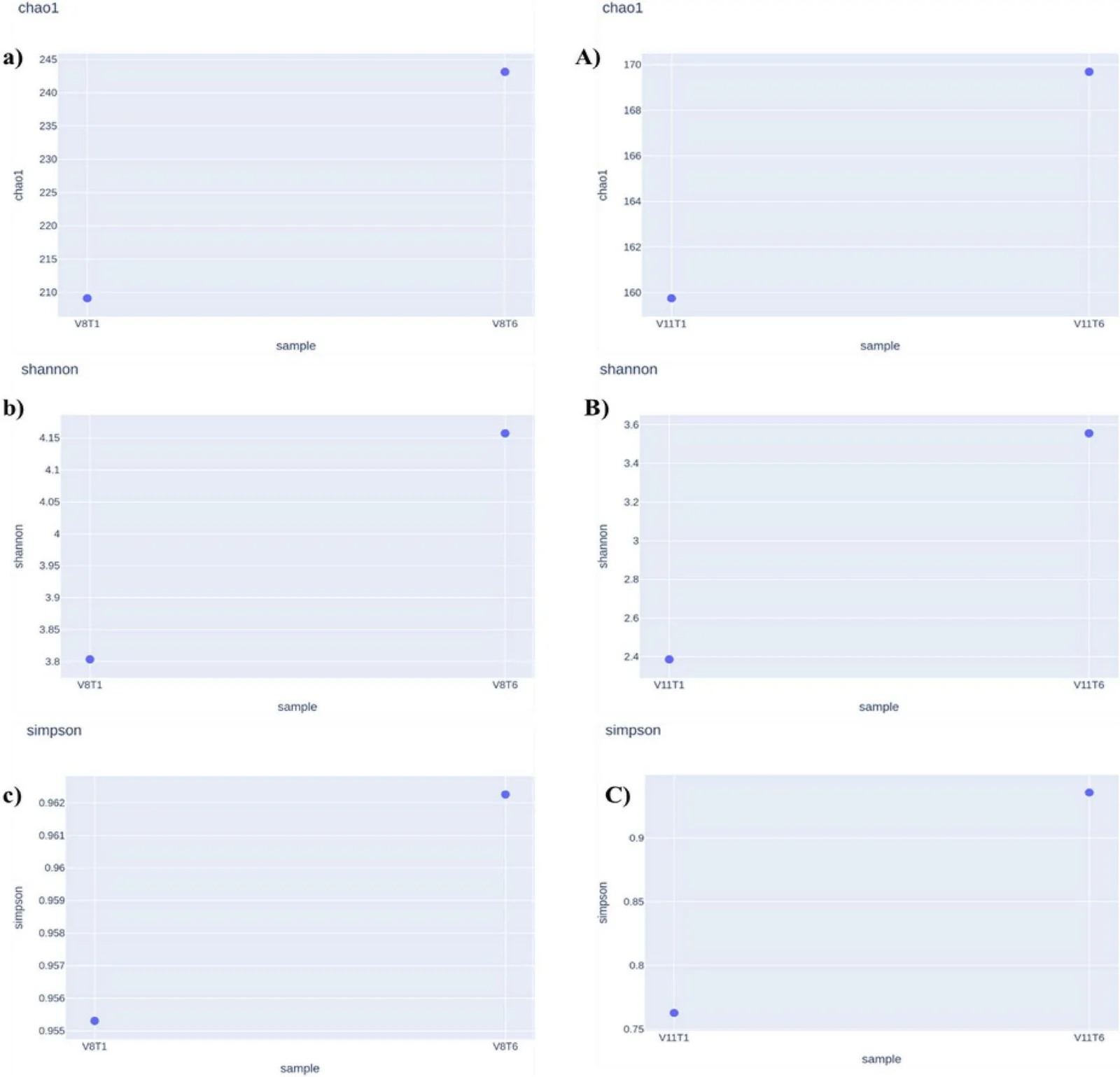
Alpha diversity indices of rhizosphere microbial communities in VBN8 and VBN11. Comparison of Chao1 (richness), Shannon (diversity), and Simpson (evenness) indices between control (T1) and consortium-treated (T6) samples at 70 DAS. **(a–c)** VBN8 and **(A–C)** VBN11. Consortium treatment increased microbial richness and diversity compared to the control.

Alpha diversity analysis showed that consortium-treated soils (T6) exhibited higher microbial richness and diversity than control (T1) in both VBN8 and VBN11 (Figure 12). Chao1 values were increased under T6, indicating greater species richness. Similarly, higher Shannon and Simpson indices in treated samples reflect enhanced community diversity and evenness. Notably, the increase was more pronounced in VBN11 compared to VBN8, indicating cultivar-specific responses to consortium application. Additionally, the consistent trend across all indices suggests a stable shift in microbial community structure under treatment conditions. Overall, the consortium application was associated with a more diverse rhizosphere microbial community under saline conditions.

### 3.14 Genus-level composition

**FIGURE 13:**
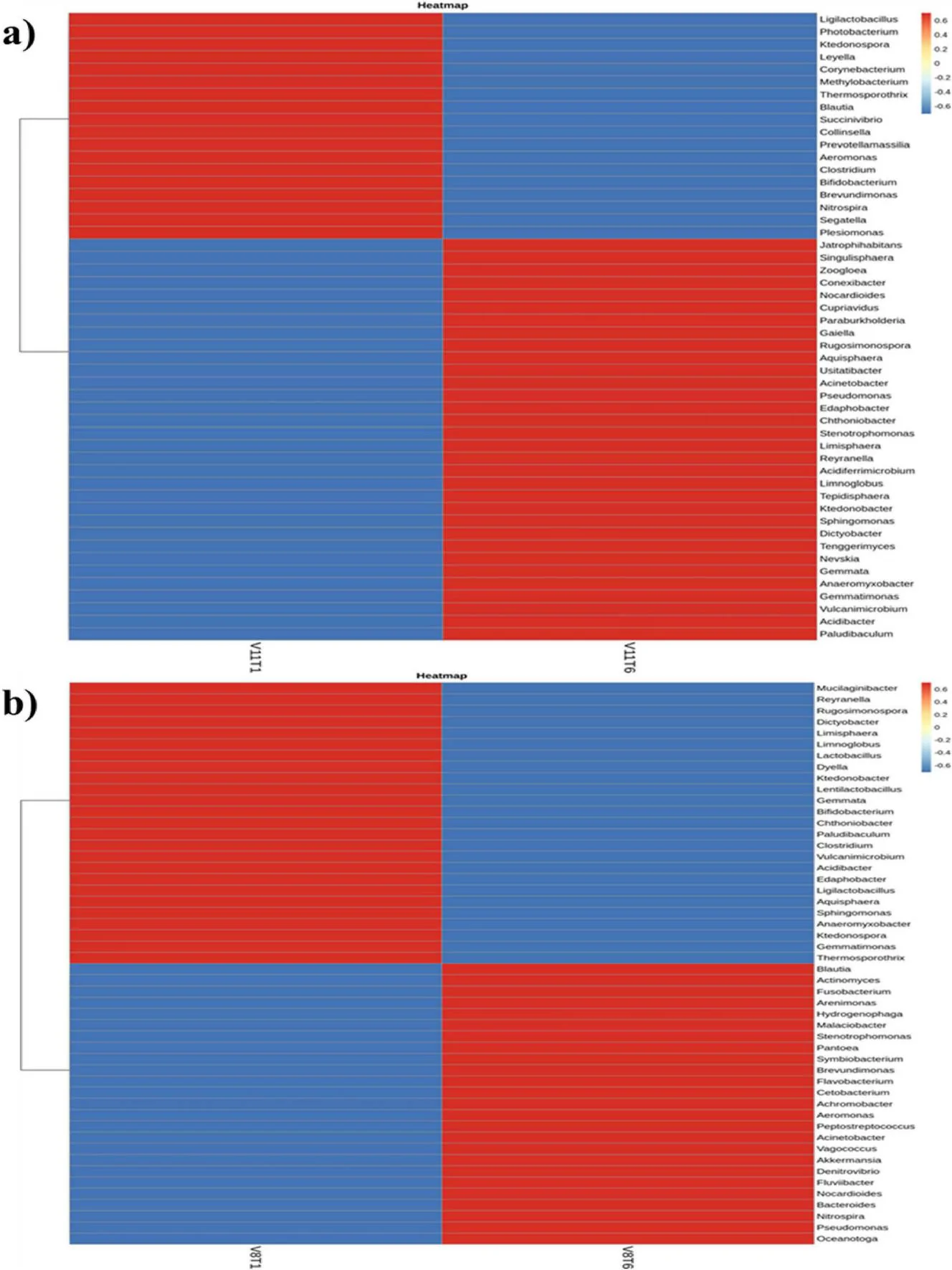
Genus-level heatmap of rhizosphere microbial communities in VBN8 and VBN11. Heatmap showing the relative abundance of dominant bacterial genera in control (T1) and consortium-treated (T6) rhizosphere soils at 70 DAS. **(a)** VBN8 and **(b)** VBN11. Red indicates higher relative abundance, while blue indicates lower abundance. Consortium treatment resulted in distinct shifts in the abundance of several bacterial genera compared to the control.

Genus-level analysis revealed distinct differences in microbial community composition between control (T1) and consortium-treated (T6) soils in both VBN8 and VBN11 (Figure 13). Several genera exhibited higher relative abundance in control samples, while others were enriched under consortium treatment, indicating a clear shift in microbial structure. In VBN8, consortium treatment promoted the enrichment of multiple genera while reducing the abundance of others present in control soils, reflecting selective modulation of the microbial community. Similarly, in VBN11, a contrasting pattern of enrichment and reduction across genera was observed between T1 and T6 samples. Overall, the heatmap highlights treatment-specific clustering and differential abundance of microbial genera.

### 3.15 Soil pH and electrical conductivity (EC)

Initial soil analysis before sowing showed similar physicochemical properties in both black gram varieties, with a soil pH of 7.6 and electrical conductivity (EC) of 1.2 dS m^−1^. After harvest, salinity treatments markedly influenced soil pH and EC values in both VBN8 and VBN11.

Among the treatments, plants exposed to 100 mM NaCl without bacterial inoculation (T3) recorded the highest EC values, reaching 6.6 dS m^−1^ in VBN8 and 6.9 dS m^−1^ in VBN11, indicating substantial salt accumulation in the rhizosphere soil. In contrast, consortium inoculation under saline conditions reduced EC values compared with uninoculated saline treatments. Under moderate salinity (T5), EC values decreased to 3.1 and 3.2 dS m^−1^ in VBN8 and VBN11, respectively, while under severe salinity (T6), EC values were reduced to 5.7 and 6.1 dS m^−1^ compared with T3.

Soil pH also showed a slight increase under salinity stress treatments, whereas consortium inoculation maintained comparatively lower pH values. The lowest EC values were observed in consortium-inoculated plants under non-saline conditions (T4), indicating improved soil conditions following bacterial inoculation (Table 3).

## 4 Discussion

The present study demonstrates that inoculation with the halophilic plant growth-promoting bacterial (HPGPB) consortium comprising MKM3, MKM4, and MKM11 substantially alleviated the adverse effects of salinity on black gram by improving plant growth, physiological performance, nutrient acquisition, antioxidant defense, and rhizosphere microbial composition. Salinity significantly reduced plant height, root length, nodulation, biomass accumulation, photosynthetic efficiency, and grain yield in both VBN8 and VBN11, with the greatest reductions observed under 100 mM NaCl. These responses are consistent with the well-established effects of salinity, where excessive Na^+^ accumulation disrupts cellular ion homeostasis, restricts water uptake, impairs photosynthesis, and accelerates oxidative damage, ultimately limiting crop productivity (Giannelli et al., 2023; Balasubramaniam et al., 2023; Liu et al., 2025). However, consortium inoculation markedly alleviated these reductions under both moderate and severe salinity, indicating that the combined activity of the three compatible halophilic strains effectively enhanced plant adaptation to saline conditions. Moreover, the improved growth observed under non-saline conditions indicates that the consortium functions not only as a stress mitigator but also as an efficient plant growth promoter.

One of the major physiological consequences of salinity observed in this study was nutrient imbalance. Salinity increased Na^+^ accumulation while simultaneously reducing the uptake of essential nutrients, including N, K, Ca, Mg, Zn, and Fe. These changes were accompanied by lower biomass production and grain yield, suggesting that ionic toxicity and nutrient deficiency jointly contributed to growth inhibition. In contrast, consortium-treated plants accumulated significantly less Na^+^ while maintaining higher concentrations of essential macro- and micronutrients. Improved nutrient uptake likely enhanced enzyme activity, membrane stability, chlorophyll biosynthesis, and overall metabolic performance, thereby contributing to greater plant productivity. Similar improvements in ionic homeostasis have been reported for halotolerant PGPR that regulate nutrient availability and maintain favorable K^+^/Na^+^ ratios under saline conditions (Kumawat et al., 2023; Al-Turki et al., 2023). In addition, consortium inoculation reduced soil electrical conductivity after harvest, suggesting that microbial activity contributed to improved rhizosphere conditions and partial alleviation of salt stress. Collectively, these findings indicate that improved nutrient acquisition and maintenance of ionic balance were major factors underlying the enhanced salinity tolerance observed in inoculated plants.

The improved nutrient status was closely associated with enhanced photosynthetic performance. Salinity markedly reduced chlorophyll content, carotenoids, stomatal conductance, transpiration rate, and net photosynthetic rate, reflecting impaired carbon assimilation under salt stress. These reductions are expected because salt-induced osmotic stress restricts stomatal opening, while ionic toxicity damages chloroplast structure and pigment stability. Consortium inoculation significantly restored pigment concentrations and gas-exchange characteristics under both salinity levels, indicating improved maintenance of the photosynthetic apparatus. The corresponding increases in biomass and grain yield demonstrate that improved photosynthetic efficiency translated directly into enhanced productivity. Similar relationships between PGPR inoculation, improved photosynthesis, and increased biomass have been reported in legumes and cereals exposed to salinity (Pan et al., 2019; Sobahan et al., 2025; Jabborova et al., 2025), supporting the physiological mechanisms observed in the present study.

Oxidative stress is one of the major constraints limiting plant growth under saline conditions. In the present study, salinity significantly increased proline and malondialdehyde (MDA) contents in both black gram varieties, indicating enhanced osmotic stress and membrane lipid peroxidation. Proline accumulation represents an adaptive response that helps maintain cellular osmotic balance, stabilizes proteins and membranes, protects enzyme activity, and scavenges reactive oxygen species (ROS) under saline conditions. The greater accumulation of proline under severe salinity reflects the increased intensity of osmotic stress. In contrast, elevated MDA content is a reliable indicator of ROS-induced membrane damage resulting from excessive lipid peroxidation under salt stress.

Salinity also markedly enhanced the activities of antioxidant enzymes, including superoxide dismutase (SOD), peroxidase (POD), catalase (CAT), and ascorbate peroxidase (APX), reflecting activation of the plant’s antioxidant defense system to counteract oxidative stress. SOD catalyzes the conversion of superoxide radicals into H_2_O_2_, whereas CAT and APX subsequently detoxify H_2_O_2_ into water and oxygen. POD further contributes to peroxide detoxification and maintenance of membrane stability. Consortium inoculation significantly enhanced the activities of these antioxidant enzymes while substantially reducing MDA accumulation, indicating more efficient ROS scavenging and improved protection against oxidative damage. Furthermore, consortium-treated plants exhibited lower proline accumulation under severe salinity despite superior growth and physiological performance, suggesting that the bacterial consortium alleviated stress severity, thereby reducing the requirement for excessive osmotic adjustment. The enhanced antioxidant capacity was associated with improved chlorophyll retention, higher photosynthetic efficiency, greater biomass accumulation, and increased grain yield, demonstrating that effective regulation of oxidative stress was a key mechanism underlying consortium-mediated salinity tolerance. Similar PGPR-mediated enhancement of antioxidant metabolism and reduction of oxidative damage under saline conditions have been widely reported (Ahmed et al., 2021; Zhao et al., 2025; Dong et al., 2024).

A notable finding of this study was the differential varietal response to consortium inoculation. Although both VBN8 and VBN11 benefited from microbial treatment, VBN11 consistently exhibited superior growth, photosynthetic activity, antioxidant responses, nutrient uptake, and yield under saline conditions. These observations suggest that the effectiveness of microbial inoculation depends partly on host genotype and that intrinsic genetic differences influence plant responsiveness to beneficial microorganisms. The greater improvement observed in VBN11 indicates that combining stress-tolerant cultivars with compatible microbial consortia may provide greater resilience than either strategy alone, an aspect that deserves further investigation in breeding and bioinoculant development programs.

Metagenomic analysis further demonstrated that consortium application was associated with distinct changes in rhizosphere microbial community composition at harvest. Consortium-treated soils exhibited enrichment of beneficial bacterial genera, including *Bacillus*, *Pseudomonas*, and *Halomonas*, together with a modest increase in microbial diversity. These taxa are well known for efficient rhizosphere colonization, nutrient cycling, phytohormone production, stress tolerance, and biofilm formation, all of which may contribute to improved plant performance under saline conditions. Although sampling was performed only at harvest and therefore cannot resolve temporal colonization dynamics or persistence of the inoculated strains, the observed community restructuring indicates that consortium inoculation influenced the assembly of the rhizosphere microbiome. These findings agree with previous reports showing that microbial inoculants improve plant performance not only through direct plant–microbe interactions but also by modifying the composition and function of native microbial communities (Berg et al., 2017; Compant et al., 2019; Egamberdieva et al., 2017). Nevertheless, because direct strain tracking was not performed, these microbiome shifts should be interpreted as associations rather than evidence of sustained colonization.

Overall, the present study demonstrates that the HPGPB consortium enhanced salinity tolerance through multiple complementary mechanisms rather than a single physiological process. Improved nutrient acquisition and ionic homeostasis reduced Na^+^ toxicity, strengthened photosynthetic capacity, enhanced antioxidant defense, and ultimately increased biomass, nodulation, and grain yield under saline conditions. Unlike many previous studies that focused primarily on single bacterial isolates or individual physiological responses, this work integrates plant growth, nutrient dynamics, physiological traits, antioxidant metabolism, soil properties, and rhizosphere microbiome analysis to provide a comprehensive understanding of consortium-mediated salinity tolerance in black gram. Nevertheless, the study has certain limitations. Colonization and persistence of the inoculated strains were not directly verified, and microbial functional predictions were based on PICRUSt inference rather than direct metagenomic functional analysis. Therefore, future studies should validate strain establishment using strain-specific molecular markers or fluorescence-based tracking, confirm predicted microbial functions through shotgun metagenomics or metatranscriptomics, investigate the regulation of stress-responsive genes and ion transporters, and evaluate consortium performance under multi-location field conditions. Such investigations will facilitate the development of reliable consortium-based bioinoculants for the sustainable cultivation of black gram in salt-affected agricultural soils.

## 5 Conclusion

This study demonstrates that a compatible halophilic plant growth-promoting bacterial (HPGPB) consortium comprising MKM3 (*Halobacillus marinus*), MKM4 (*Halobacillus halophilus*), and MKM11 (*Halobacillus halophilus*) effectively enhanced salinity tolerance in black gram under both moderate (50 mM NaCl) and severe (100 mM NaCl) salinity stress. Consortium inoculation significantly improved plant growth, nodulation, biomass accumulation, grain yield, photosynthetic performance, nutrient uptake, and antioxidant defense while reducing Na^+^ accumulation, oxidative damage, and soil electrical conductivity compared with uninoculated plants. The superior response of VBN11 relative to VBN8 further highlights the importance of integrating stress-tolerant cultivars with compatible microbial inoculants to maximize crop performance in saline environments.

Beyond improving plant physiology, the consortium was associated with distinct shifts in rhizosphere microbial community composition, suggesting that microbiome modulation contributes to enhanced plant adaptation under salinity stress. By integrating physiological, biochemical, nutrient, soil, and rhizosphere metagenomic analyses, this study provides comprehensive evidence that consortium-based HPGPB inoculation enhances salinity tolerance through coordinated interactions between plants and the microbiome rather than through a single stress-mitigation mechanism. Overall, the findings establish this multi-strain HPGPB consortium as a promising, environmentally sustainable bioinoculant to improve black gram productivity in salt-affected soils and advance climate-resilient agriculture. Future studies should validate its field performance across diverse agroecological conditions, assess formulation stability and long-term persistence, and investigate the molecular and functional mechanisms governing plant–microbiome interactions to facilitate its large-scale agricultural application.

## Data Availability

The datasets presented in this study can be found in online repositories. The names of the repository/repositories and accession number(s) can be found in the article/Supplementary material.
