# Supplementary material for: Halophilic plant growth-promoting bacterial consortium reshapes soil microbiota to enhance salinity tolerance, antioxidant defense, and yield in Vigna mungo L
Source: Front Microbiol. 2026 Aug 11;17:1878014. doi: 10.3389/fmicb.2026.1878014 (PMC13504803; doi:10.3389/fmicb.2026.1878014)
Supplement: Supplementary file 1 [file Supplementary_file_1.docx]

**Figure S1.** Species-level microbial community composition in VBN8 and VBN11 rhizosphere soils. Stacked bar plots showing the relative abundance (%) of dominant bacterial phyla in control (T1) and consortium-treated (T6) soils at harvest (70 DAS). (A) VBN8 and (B) VBN11. Each colored segment corresponds to an individual bacterial species, illustrating variations in microbial community structure among the treatments.

**Figure S2.** Core microbiome analysis showing the prevalence of dominant bacterial taxa across different detection thresholds in (a) VBN8, and (b) VBN11 pot soil samples. Heatmap colors represent the prevalence (%) of core taxa detected at varying relative abundance thresholds. Red indicates high prevalence, whereas blue indicates low prevalence. *Clostridium*, *Pseudomonas*, and *Bifidobacterium* were identified as major core taxa across the analyzed samples, demonstrating their consistent occurrence within the microbial communities.

**Figure S3.** KEGG functional pathway prediction of microbial communities associated with (a) V8T1, (b) V8T6, (A) V11T1, and (B) V11T6 samples based on PICRUSt analysis of 16S rRNA sequencing data. Functional categories were classified into Level-1 groups, including Cellular Processes, Environmental Information Processing, Genetic Information Processing, Metabolism, and Organismal Systems. The bar plots represent the relative abundance of predicted Level-2 functional pathways across the different samples, indicating variations in metabolic and cellular functional potentials among the microbial communities.
